# *Aedes aegypti* strain selected with *Bacillus thuringiensis* svar. *israelensis* larvicide for 50 generations remains susceptible and exhibited increased fitness

**DOI:** 10.1186/s13071-025-07037-x

**Published:** 2025-10-07

**Authors:** Heverly Suzany Gouveia Menezes, Louise Helena Guimarães Oliveira, Ana Paula Araújo, Karine Silva Carvalho, Maria Helena Neves Lobo Silva-Filha

**Affiliations:** 1 Department of Entomology, Instituto Aggeu Magalhães-Fiocruz, Av. Moraes Rego S/N, Recife, PE 50740-465 Brazil; 2 Instituto Federal da Paraíba, Sousa, PB 58814-000 Brazil

**Keywords:** Bti, Cry receptors, Resistance, Reserves, Longevity, Quiescence

## Abstract

**Background:**

*Bacillus thuringiensis* serovar *israelensis* (Bti) was the first bacterial larvicide developed for dipteran control. Its insecticidal crystal, composed of protoxins such as Cry11Aa, Cry4Ba, Cry4Aa, and Cyt1Aa, exhibits a selective and complex mode of action and a low potential for resistance development. Most resistance studies have focused on populations from regions where larvicide applications are seasonal, while *Aedes aegypti* populations in tropical endemic areas may face continuous selection pressure, raising concerns about potential selection of resistance. This study evaluated an *Ae. aegypti* strain (RecBti), Subjected to strong Bti selection for 50 generations under laboratory conditions, as part of a longitudinal study for assessing in vivo susceptibility to Bti and also biological traits that could be potentially influenced by this chronic larvicide exposure.

**Methods:**

The RecBti strain, continuously selected using a commercial Bti larvicide for 50 generations, was compared with reference strains. Bioassays with Bti, Cry11Aa, and Cry4Ba were performed to assess the susceptibility of larvae (F_40_ and F_50_) to these compounds. Reverse transcription quantitative polymerase chain reaction (RT-qPCR) assays were carried out to investigate the expression of midgut-bound proteins reported as Cry toxin receptors in F_50_ larvae. Energy reserves (lipids and reducing sugars) were quantified in larval pools and adult females from F_50_. Additionally, biological parameters of egg viability after varying quiescence periods and adult longevity, were also evaluated in F_50_.

**Results:**

RecBti larvae remained susceptible to the Bti crystal and its individual Cry11Aa and Cry4Ba toxins after 50 generations of chronic exposure, compared with the reference susceptible strain. The transcriptional analysis revealed that three genes encoding Cry midgut receptors—aminopeptidase, alkaline phosphatase, and cadherin—were expressed at significantly higher levels in RecBti larvae compared with the reference strain. RecBti larvae and adults exhibited significantly higher lipid reserves, although no significant difference in reducing sugar levels was observed compared with the reference individuals. Egg viability of RecBti females was higher, and adults showed increased longevity.

**Conclusions:**

This study provides evidence for the low risk of resistance development of *Ae. aegypti* to Bti, on the basis of a prolonged and intensive selection process. The levels of expression of Cry toxin receptors found in RecBti larvae, compared with reference larvae, are consistent with the status of in vivo susceptibility. However, long-term Bti exposure was associated with physiological and biological changes that may have implications for mosquito fitness and vectorial competence. These findings show that Bti is the larvicide that has a low potential to select resistance, compared with other compounds available, and highlight the importance of monitoring other effects of larvicide on the biology of mosquitoes.

**Graphical Abstract:**

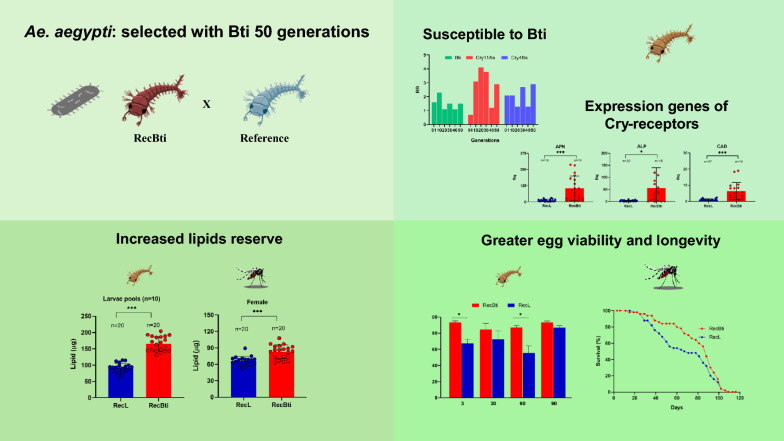

**Supplementary Information:**

The online version contains supplementary material available at 10.1186/s13071-025-07037-x.

## Background

*Aedes aegypti* is the primary vector of several arboviruses in the Americas, and the global burden of arboviral diseases transmitted by *Aedes* species continues to rise [1]. Dengue reached a historic peak in 2024 in Brazil, with more than 6.5 million cases reported [2]. Controlling *Aedes* populations remains a global challenge, particularly in low- and middle-income countries where arboviruses are endemic. Among the tools used in integrated vector control programs, microbial larvicides play an important role [3, 4]. *Bacillus thuringiensis* serovar (svar.) *israelensis* (Bti)-based larvicides, which contain insecticidal crystals, were introduced worldwide in the 1980s for the control of dipteran larvae [5, 6] and remains one of the safest larvicides among those commercially available [7]. Its specific and high activity against dipteran larvae, including *Aedes* spp., occurs owing to a synergistic interaction among the insecticidal components of the crystal: three crystal (Cry) protoxins from the three-domain family (Cry11Aa, Cry4Ba, and Cry4Aa) and one cytolytic protoxin (Cyt1Aa) [8, 9].

The insecticidal action of the Bti-based larvicides depends on ingestion of the crystals, followed by proteolytic activation of protoxins into active toxins that bind specifically to receptors in the larval midgut [9]. Several midgut proteins have been reported as receptors, including aminopeptidases (APNs), alkaline phosphatases (ALPs), α-glucosidases (GLUs), cadherins (CADs), and adenosine triphosphate (ATP)-binding cassette (ABC) transporters [10, 11]. Although additional molecules and mechanisms may contribute to Bti toxicity, the binding of Cry toxins to these midgut receptors is considered the primary mechanism of action. For that, the Cyt1Aa enhances the activity of Cry toxins by acting as a receptor that induces conformational changes and the formation of Cry oligomers, which have a high-affinity binding to midgut receptors of larvae [12–14]. This unique mode of action is considered less vulnerable to mechanisms of resistance owing to target-site alterations.

After decades of use, no cases of resistance to larvicides containing the Bti crystals have been reported in the field [7, 15–18]. Laboratory efforts to select *Ae. aegypti* resistance were successful only when individual Cry toxins were used [19–22], while selection with the full Bti crystal has not yielded resistant strains [21, 23–26]. Nevertheless, resistance monitoring remains essential, as many studies have been conducted in countries where larvicide applications are seasonal, potentially interrupting consistent selection pressure. Susceptibility to Bti needs to be evaluated considering the scenario of areas where *Aedes* populations are permanently established with high population densities and larvicides are applied year-round [27]. Assessments of *Aedes* susceptibility to several larvicides available have shown that larvae from different geographic areas are susceptible to Bti, contrasting with the records of resistance to other tested compounds, and highlighting the role of Bti to manage resistance [28–35]. It is worth noting that susceptibility studies that do not provide previous information about the background of previous exposure of larvae and do not use reference mosquito strains or insecticide reference standards for the consistency of data analysis might lead to misunderstood conclusions [36–39].

We hypothesize that Bti-based larvicides have a low potential for selecting resistance, owing to their unique mode of action, compared with other compounds available. Nonetheless, long-term investigations under controlled selection procedures are necessary to enhance our understanding of the risk of resistance development to Bti. To address this issue, we previously established an *Ae. aegypti* strain (RecBti), under insectary conditions, that has been Subjected to selection pressure using a widely deployed commercial Bti-based larvicide. The last assessment, after 35 generations of selection, revealed no evidence of resistance to Bti [40]. Among the biological traits of F_35_ individuals that were investigated, no significant changes were detected, except for the increased female susceptibility to the Zika virus [41]. The present study aimed to assess the RecBti strain after 50 continuous generations of Bti selection, as part of this longitudinal evaluation of susceptibility, in addition to other biological parameters relevant to vector competence that may be influenced by long-term Bti exposure.

## Methods

### *Aedes aegypti* strains

A total of three *Ae. aegypti* strains were used in this study: Rockefeller (Rocke) reference strain, RecL reference strain, and RecBti test strain. The Rocke strain has been maintained in the insectary of the Institute Aggeu Magalhães (IAM)-Fiocruz since 2007 and was used as the international reference strain for monitoring larvicide susceptibility in bioassays. The RecL is a local strain, originating from Recife city as RecBti, that has been maintained in the insectary without exposure to any control agent since 1996 [42]. The RecL was used as a control strain for the other evaluations. The RecBti strain was established with eggs from Recife city (Brazil), and larvae have been selected for 50 generations with Bti, as described below. All strains have been maintained under insectary-controlled conditions (26 ± 1 °C, 70% relative humidity, 14 h:10 h light:dark photoperiod). Larvae were reared in dechlorinated tap water and fed commercial cat food (Friskies*®*), while adults were provided with a 10% sucrose solution. Females received defibrinated rabbit blood via an artificial feeding system for 1 h at 37 °C, once per week.

### Bti compounds

The samples used for evaluating the susceptibility in bioassays were: IPS-82 lyophilized reference powder (Institut Pasteur, Paris) containing crystals/spores from a sporulated whole culture (H14 serotype) with 15,000 international toxic units/mg (ITU) and Cry11Aa and Cry4Ba lyophilized powders (IAM-Fiocruz) containing crystals/spores of each recombinant protoxin obtained from sporulated cultures of the Bti 4Q2-82 acrystaliferous strain [43]. The toxicity of Cry11Aa and Cry4Ba toxins was assessed since they display the highest individual larvicidal activity among the protoxins from the Bti crystal. The Bti-based larvicide used for the selection of RecBti larvae was Vectobac*®* WG (Valent BioSciences, Illinois, USA), containing 37.4% crystals/spores of Bti H-14 (AM6552 strain) with a potency of 3000 ITU.

### Selection of the RecBti strain

The RecBti parental strain was established using large and diverse eggs samples collected in 40 neighborhoods of Recife city. A total number between 5000 and 14,500 third-instar larvae per generation was exposed to a Bti-based larvicide Vectobac*®* WG (Valent BioSciences, Illinois, USA) with concentrations that provoked at least 60% mortality after 24 h exposure. The selection procedures are fully described in Carvalho et al. [23]. The susceptibility of larvae to Bti and its toxins has been evaluated throughout the selection process, with the last assessment conducted using F_35_ larvae [40].

### In vivo susceptibility assays

In the present study, we evaluated the susceptibility of RecBti larvae from two generations (F_40_ and F_50_) to Bti and its protoxins. For that, dose–response bioassays were performed to determine the lethal concentrations (LC) for 50% (LC_50_) and 90% (LC_90_) of Bti, and the LC_50_ of Cry11Aa and Cry4Ba protoxins to third-instar larvae after a 24-h exposure period, as previously described [44]. Briefly, experimental groups of third-instar larvae (*n* = 20), placed in 100 mL tap water, were treated with a range of five to seven concentrations able to provoke between 10% and 100% mortality after the exposure. For each larvicidal compound, a stock aqueous suspension of lyophilized powders containing spores–crystals (5 g/L) was prepared with distilled water, and aliquots were stored at −20 °C. This stock Suspension was used to prepare the dilutions needed for the corresponding concentrations. All experimental groups were tested in triplicate, including an untreated control group. Mortality was recorded after 24 h. Bioassays that showed > 10% mortality in the untreated control group were excluded from the analysis. Lethal concentrations were determined through Probit analysis (SPSS, version 16.0, for Windows and Statistics 25). Resistance ratios (RR) between the LC to the RecBti strain and the respective LC to the Rocke reference strain for insecticide susceptibility were determined. RR values below tenfold were considered natural variations, as proposed by Araujo et al. [28], on the basis of the dataset of Bti susceptibility of untreated populations available in literature.

### RT-qPCR assays

The relative quantification of transcripts of genes encoding proteins previously reported as receptors for the Bti toxins was performed using quantitative reverse transcription polymerase chain reaction (RT-qPCR) assays. Three *Ae. aegypti* genes indicated as receptors of Cry11Aa, the most active toxin from Bti [45–49], were selected to be investigated: aminopeptidase (ID no. AAEL012778), alkaline phosphatase (ID no. AAEL015070), and cadherin (ID no. AAEL007478). The relative quantification of these transcripts in the control larvae (RecL) was compared with RecBti larvae (F_50_). RNA was extracted from pools of ten third-instar larvae using Trizol™ reagent (Thermo Fisher Scientific), according to the manufacturer’s instructions, and the RT-qPCR was performed using QuantiTect*®* SYBR Green RT-PCR*®* Kit (Qiagen). Specific primers for the target genes and for the ribosomal protein *18S*, used as the endogenous control gene, are provided in Additional file 1: Supplementary Table S1. Reactions were run on ABI 7500*®* (Applied Biosystems, Waltham, MA, USA), and the relative quantification was calculated using the 7500-software, version 2.3, with the relative quantification (ΔΔCT) module (Applied Biosystems). The cycle threshold (CT) of a RecL replicate, whose value was the closest to the average of the CT values of the replicates of this control strain, was used as the reference sample [50].

### Assays for lipids quantification

The amount of total lipids was quantified using a vanillin-phosphoric acid colorimetric method that has been previously described [51]. The comparison of individuals from the RecBti strain (F_50_) and the RecL strain was done using pools of ten early third-instar larvae or newly emerged (until 20 h) individual females, without access to the sucrose diet, and processed as described above. The absorbance of the samples was recorded at 525 nm in the UltroSpec2100*™* (Amersham Biosciences, Amersham, UK). The amount of lipids in the samples was determined using a standard curve (25–300 µg) of commercial soybean oil (Soya*®* batch L425B, São Paulo, SP, Brazil), mainly composed of triglycerides, which was read under the same conditions as the test samples.

### Assay for reducing sugar quantification

The quantification of reducing sugars was based on the protocol of Yamada et al. [52] using pools of twenty early third-instar larvae and newly emerged individual females (20 h) without access to the sucrose diet. Individuals of RecBti (F_50_) and RecL strains were compared. Samples were homogenized with methanol, ultrapure water, and chloroform (200 µL, 2:1:1) under ice, incubated (−30 °C, 30 min), and centrifuged (21,000 × *g*, 4 °C, 20 min). The pellet was washed with ethanol (300 µL) and dried at room temperature (RT). The reagents used had a purity ≥ 99.8%. Phosphate saline buffer (0.2X, pH 7.4) with Triton X-100 0.1% (400 µL) was added to the sample and incubated (70 °C, 30 min). After this, the sample was split into a test sample (200 µL) and a negative control sample (200 µL). The test sample was incubated with amyloglucosidase (0.5 mg/mL final, Sigma-Aldrich) at 60 °C for 1 h. The negative control sample was incubated under the same conditions without that enzyme. Thereafter, dinitrosalicylic acid (500 µL) was added to the samples, the reaction was stopped by heating (100 °C, 6 min), and the samples were incubated on ice for 15 min. The absorbance of the test and control samples was read at an absorbance of 540 nm in a 96-well microplate reader, Benchmark Plus*™* (Bio-Rad, Hercules, CA, USA). The absorbance was converted to glucose concentration (Sigma-Aldrich) on the basis of a standard curve (0.06–3 mg/mL). The concentration of reducing sugars was determined on the basis of the variation of the glucose concentration observed in the test sample and the negative control sample, and the amount determined in the sample was used.

### Assessments of egg viability and adult longevity

To assess these traits, individuals from RecBti (F_50_) and RecL strains were reared and kept under controlled insectary conditions. To produce adults, oviposition papers containing eggs were submerged in tap water with grass infusion (6 g/L) to stimulate hatching of first-instar larvae. Samples of 100 first-instar larvae, collected within 24 h after hatching, were transferred to plastic trays (20 cm length × 16 cm width × 8 cm depth, 2 L capacity) filled with 1 L of tap water and cat food (Friskies*®*), provided on days 0 (100 mg), 4 (150 mg), and 6 (300 mg) during the larval phase. After the emergence, experimental groups of 25 females and 25 males (1:1 ratio) were set in a plastic cage (12 cm height × 10 cm diameter) with 10% sucrose solution and water ad Libitum, refreshed twice per week. For assessing egg viability, a blood meal was offered to 5-day-old females. A total of three days after the blood meal, two plastic recipients, each with water (50 mL) and two sections of oviposition paper (7 cm length × 5 cm width), were placed in the cages as Substrates for female oviposition for 2 days. After that, the papers with eggs were collected, allowed to complete the embryogenesis in a humid chamber for 72 h, and then stored at RT for 180 days. The viability of eggs was evaluated using samples of at least 80 eggs, with different periods of quiescence: 3, 30, 60, 90, and 180 days. For each time point, the number of eggs in the samples (*n* > 80) was recorded under a stereoscope, in quadruplicate. For inducing larvae, hatching egg samples were set in a Petri dish with 25 mL spring water with grass infusion (6 g/L). First-instar larvae were recorded and removed daily from the dish, until 96 h. Two independent assays were carried out.

For determining longevity, similar experimental groups of adults (25 females: 25 males) kept with a 10% sucrose solution and water ad Libitum were monitored. No blood meal was offered to those groups. The longevity of adults in two or three replicates per strain was recorded twice per week for 120 days, or until all adults were dead. The longevity was evaluated on the basis of two independent assays.

### Statistics

Statistical analyses were performed using GraphPad Prism (version 8.0; GraphPad Software Inc., San Diego, CA, USA) and RStudio (version 12.0; R Core Team, 2024). For all analyses, data distribution was first assessed using the Shapiro–Wilk normality test. When the data did not follow a normal distribution, differences between the RecBti and RecL strains were evaluated using the non-parametric Mann–Whitney *U* test. When the data followed a normal distribution, an unpaired *t*-test with Welch’s correction was applied. Analyses of egg viability and longevity were conducted in R using the Wilcox.test function and p.adjust function. Statistical significance was determined using the Holm–Sidak method for multiple comparisons correction (*α* = 0.05). A *P*-value < 0.05 was considered statistically significant for all tests. Statistical data are provided in the corresponding additional files.

## Results

### Susceptibility to Bti, Cry11Aa, and Cry4Ba toxins

**Fig. 1:**
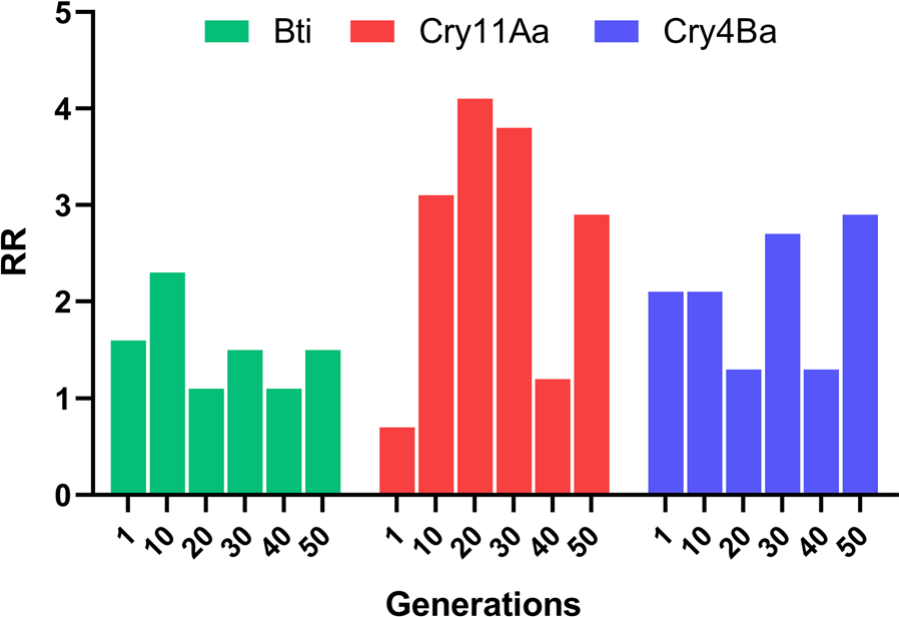
Summary of resistance ratio (RR) values of third-instar *Aedes aegypti* RecBti larvae to *Bacillus thuringiensis* svar. *israelensis* (Bti) and its protoxins (Cry11Aa and Cry4Ba) during 50 generations of selection. RR values from F_1_, F_10_, F_20_, and F_30_ were obtained from Carvalho et al. [23]

The RecBti strain was selected using a commercial Bti-based larvicide for 50 generations (Additional file 2: Supplementary Table S2). This long-term exposure involved more than 435,000 larvae throughout the process. At each generation, an average of 8500 third-instar larvae were treated with Bti-larvicide, achieving mortality rates between 60% and 91%. Each parental generation was composed of around 2000 adults that survived the treatments carried out during the larval phase. In this study, the susceptibility of RecBti larvae, from the F_40_ and F_50_ generations, to the whole Bti crystal and to Cry11Aa and Cry4Ba individual protoxins was assessed (Table 1). Larvae from both generations remained susceptible to Bti, on the basis of a comparison of LC values and their confidence intervals relative to those of the Rocke reference strain. Resistance ratios (RRs) at LC_50_ and LC_90_ for both generations were less than twofold, indicating no significant increase, after 50 generations of exposure. Regarding the individual protoxins, a slight increase in RR at LC_50_ was observed in RecBti larvae from F_50_, which reached 2.9 fold. A broader view of RR values recorded along the selection process from F_1_ to F_50_, including previous data from Carvalho et al. [23] and actual data, showed no increase of RR for Bti and for the individual toxins (Fig. 1). Some slightly higher RR values were detected in some generations, but no trend of increase was detected. The LC values presented in Table 1 are the mean of three biological replicates, and the complete dataset is available in Additional file 3: Supplementary Table S3.

**Table 1:** Toxicity of *Bacillus thuringiensis* svar. *israelensis* (Bti) and its protoxins to third-instar *Aedes aegypti* larvae from RecBti and the Rockefeller (Rocke) strains

Samples	Strain	*N*	LC_50_ (95% CI)^a^	RR^b^	LC_90_ (95% CI)	RR
F_40_						
Bti	Rocke	1200	0.014 (0.013–0.016)	1	0.033 (0.028–0.041)	1
	RecBti	1380	0.016 (0.014–0.019)	1.1	0.036 (0.031–0.046)	1.1
Cry11Aa	Rocke	1860	1.051 (0.803–1.422)	1	ND^c^	
	RecBti	1380	1.232 (0.927–1.722)	1.2	ND	
Cry4Ba	Rocke	1440	0.907 (0.622–1.344)	1	ND	
	RecBti	1200	1.178 (0.808–1.849)	1.3	ND	
F_50_						
Bti	Rocke	1020	0.009 (0.007–0.011)	1	0.023 (0.019–0.033)	1
	RecBti	1560	0.016 (0.014–0.018)	1.5	0.031 (0.027–0.037)	1.3
Cry11Aa	Rocke	1620	0.307 (0.206–0.433)	1	ND	
	RecBti	1200	0.906 (0.617–1.253)	2.9	ND	
Cry4Ba	Rocke	1080	0.553 (0.378–0.789)	1	ND	
	RecBti	720	1.599 (0.910–3.192)	2.9	ND	

^a^Lethal concentration (mg/L) for 50% and 90% of larvae exposed for 24 h (mean and 95% confidential intervals)

^b^Resistance ratio between the LC for the test strain and that for the reference strain

^c^Not determined

### Expression of midgut Cry receptors

**Fig. 2:**
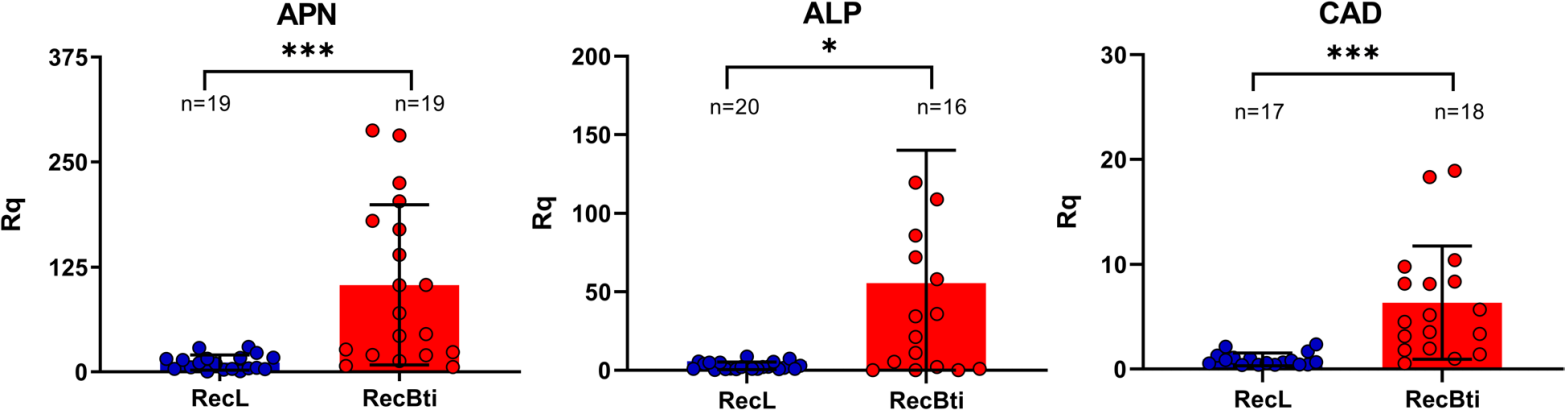
Relative quantification (Rq) of transcripts of *Aedes aegypti* genes in pools of ten third-instar larvae from a strain exposed to *Bacillus thuringiensis* svar. *israelensis* for 50 generations (RecBti) compared with a reference strain (RecL). APN-aminopeptidase (ID no. AAEL012778), ALP-alkaline phosphatase (ID no. AAEL015070), and CAD-cadherin (ID no. AAEL007478). Columns and bars represent mean and standard deviation, respectively, of biological replicates (*n* = 16–20 larvae pools) tested in duplicate. Mann–Whitney comparisons: ^***^P < 0.001, ^*^P < 0.05) statistically different (APN *P* = 0.0005; ALP *P* = 0.0156; CAD *P* = 0.0001)

To compare the expression of Cry toxin receptors in the midgut of larvae from the RecBti and the RecL control strain, we performed relative quantification of transcripts from three genes: aminopeptidase (APN AAEL012778), alkaline phosphatase (ALP AAEL015070), and cadherin (CAD AAEL007478), in pools of ten third-instar larvae of each strain (*n* = 16–20). Transcripts were detected for all genes in both strains, and APN showed the highest abundance, followed by ALP and CAD (Fig. 2). Some within-strain variation was observed. When comparing strains, RecBti larvae exhibited a higher relative quantification of APN (103.5 ± 95.5, *P* = 0.0005), ALP (55.56 ± 81.77, *P* = 0.0156), and CAD (5.3 ± 4.7, *P* = 0.0001), compared with the RecL reference strain (APN 31.4 ± 54.3; ALP 3.0 ± 2.4; CAD 2.5 ± 5.9). These findings indicate that the midgut of RecBti larvae has higher transcript levels of these potential Cry toxin receptors compared with the reference strain. The complete dataset of RT-qPCR assays is available in Additional file 4: Supplementary Table S4.

### Energy reserves

**Fig. 3:**
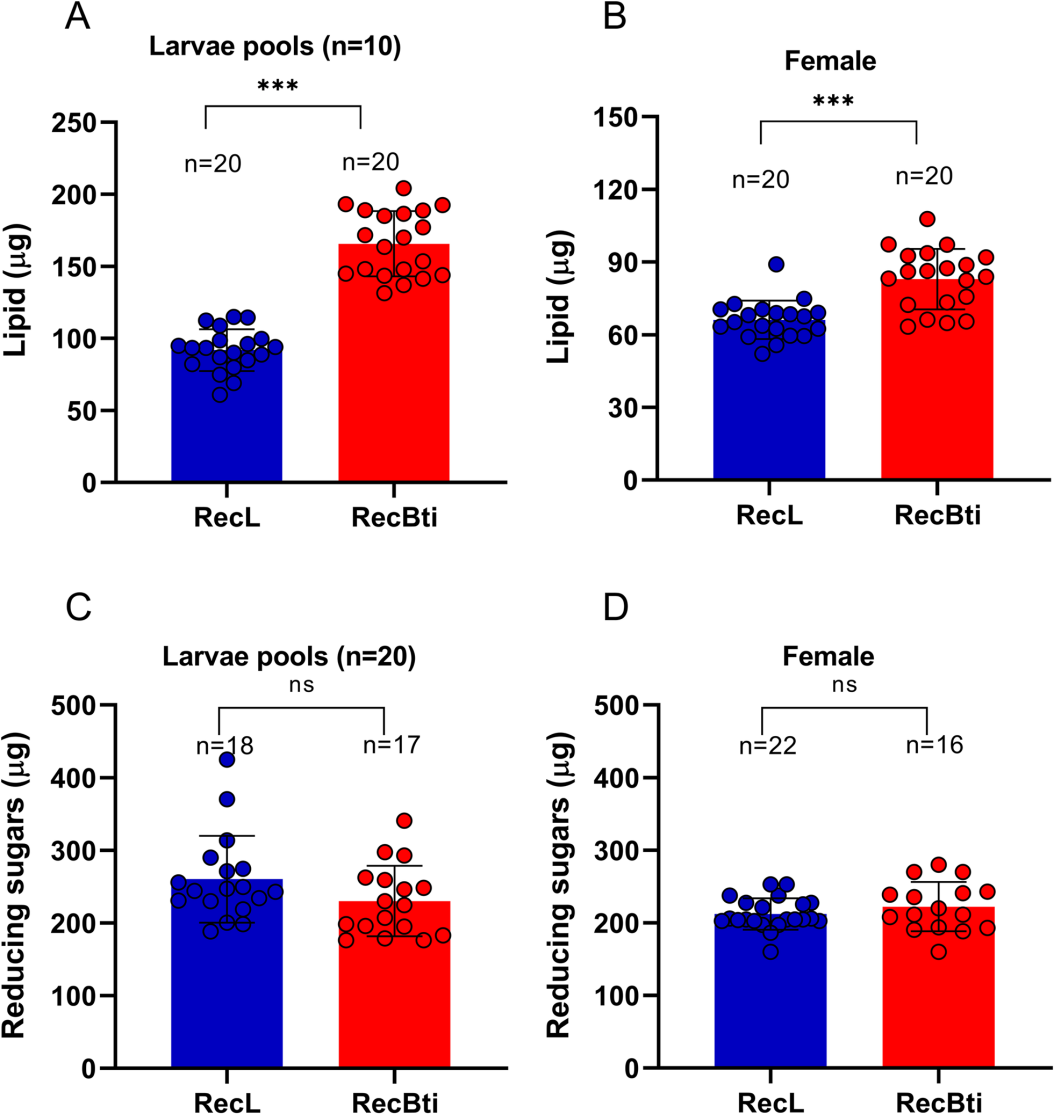
Energy reserves of *Aedes aegypti* from a strain exposed to *Bacillus thuringiensis* svar. *israelensis* for 50 generations (RecBti) compared with a reference one (RecL). **A** Lipids in pools of ten third-instar larvae. **B** Lipids in individual newly emerged females. **C** Reducing Sugars in pools of 20 third-instar larvae. **D** Reducing sugars in newly emerged females. Columns and bars represent mean and standard deviation, respectively, of biological replicates (*n* = 16–22). Unpaired *t*-test and Mann–Whitney test comparisons: ns. not statistically different, ^***^statistically different (*P* = 0.0001)

To evaluate whether the chronic exposure to Bti affected energy storage in mosquitoes, which are critical for development and survival, we quantified lipid and reducing sugar levels in RecBti strain and the RecL control strain (Fig. 3). The lipid amount in pools of ten third-instar RecBti larvae was greater (165.6 ± 22.1 µg) compared with that found in RecL larvae pools (91.9 ± 14.1 µg; *P* = 0.0001). Similarly, individual RecBti females showed a significantly greater lipid reserve (82.9 ± 12.1 µg) than RecL females (66.1 ± 7.7 µg; *P* = 0.0001). The reserves of reducing Sugars were similar for larvae and adults between strains. The average reducing sugar content in pools of 20 larvae was 230.2 ± 47.2 µg for RecBti and 260.3 ± 58.2 µg for RecL. RecBti individual females had 222.2 ± 32.8 µg and RecL females had 212.1 ± 21.1 µg. These findings suggest that prolonged Bti exposure is associated with increased lipid accumulation, while no impact on reducing sugar reserves was detected either in larvae or adults. The complete dataset is available in Additional file 5: Supplementary Table S5.

### Egg viability and adult longevity

**Fig. 4:**
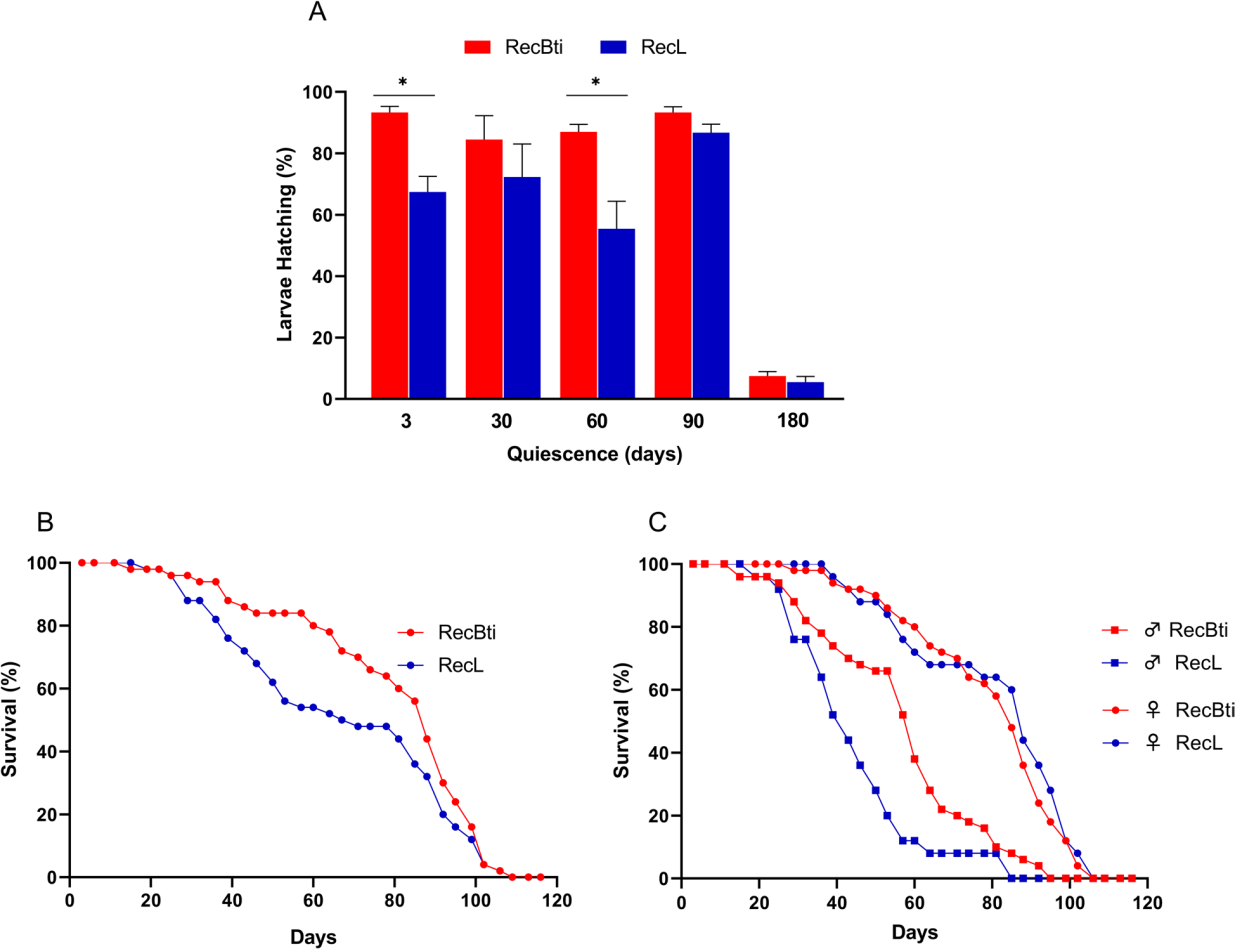
Biological parameters of an *Aedes aegypti* strain exposed to *Bacillus thuringiensis* svar. *israelensis* for 50 generations (RecBti) compared with a reference strain (RecL). **A** Viability of egg samples stored between 3 and 180 days. Replicates of egg samples (*n* > 80) were submerged in water with grass infusion (6 g/L), and first-instar larvae were recorded daily until 96 h. Columns and bars show the mean and standard deviation, respectively, of eight biological replicates (*n* = 4198 RecBti eggs, *n* = 4037 RecL eggs) after 24 h. Mann–Whitney *U* comparisons corrected using the Holm–Sidak method (*α* = 0.05): ns. not statistically different, ^*^statistically different (3 days *P* = 0.001, 60 days *P* = 0.004). **B** Longevity of adults combining data from two independent assays. **C** Longevity of adults stratified by sex. Replicates were groups of 50 adults (1:1 sex ratio). Each time point represents the mean of six (300 RecBti adults) and five (250 RecL adults) biological replicates from two assays. Mann–Whitney *U* test comparisons: significant differences were observed between days 37 and 56 (*P* < 0.05, values available in Additional file 7: Supplementary Table S7)

To investigate whether the greater Lipid reserve found in RecBti individuals would impact egg viability and adult longevity, individuals were compared with the RecL reference ones. Hatching rate of eggs produced by blood-fed females from both strains and stored for different quiescence periods was determined. A sample of more than 4000 eggs per strain was investigated in two independent assays. RecBti eggs from all timepoints, except 180 days, showed viability greater than 80% (Fig. 4A). RecBti larvae hatching was greater at each of the five quiescence timepoints analyzed, being statistically significantly at timepoint 3 days (24 h; *P* = 0.001), 30 days (96 h; *P* = 0.01), and 60 days (24 h; *P* = 0.004) (Fig. 4A, Additional file 6: Supplementary Table S6). For both strains, the viability of eggs stored for 90 days was still greater than 50% but after 180 days, it decreased sharply to less than 20% (Fig. 4A).

To assess the adult longevity, the mortality of RecBti and RecL was recorded in a replicate of 50 individuals (1 female: 1 male ratio). Two independent assays using a total sample of 300 RecBti and 250 RecL individuals were monitored until 113 days during the first assay and until 109 days in the second assay, when mortality reached 100%. The combined dataset from both assays is presented in Fig. 4, while the dataset from each assay is available in Additional file 7: Supplementary Table S7. Overall, the RecBti adults displayed a trend toward greater longevity compared with RecL, and the Survival was significantly greater between days 36 and 57 (Fig. 4B, Additional file 7: Supplementary Table S7). After 50 days, for instance, RecBti displayed 76% Survival against 32% for RecL. When analyzed by sex, males from both strains exhibited higher mortality than females, as expected (Fig. 4C). The comparison of strains showed that RecBti males had a greater survival compared with their RecL counterparts, being statistically significant during the period signaled above, while survival was similar for females from these strains (Additional file 7: Supplementary Table S7). In summary, the comparisons of these parameters showed that RecBti F_50_ individuals showed increased fitness with a greater egg viability and a trend toward greater longevity.

## Discussion

The *Ae. aegypti* RecBti strain was established to investigate whether long-term exposure to a commercial Bti-based larvicide could lead to the selection of resistance to this compound. Our data demonstrated that, after 50 generations under strong and continuous selection pressure in controlled insectary conditions, RecBti larvae remained Susceptible to Bti insecticidal crystal. This finding corroborates previous assessments of this strain, which also reported no evidence of resistance onset until 35 generations of exposure [23, 41]. To our knowledge, this is the longest and most intensive laboratory-based selection experiment demonstrating a sustained lack of resistance to Bti in *Ae. aegypti*. This study was designed to simulate conditions of endemic regions where larvicide treatments are applied continuously throughout the year, in contrast to settings where seasonal applications may interrupt consistent selection pressure and underestimate the potential for resistance development under continuous exposure. One of the most prominent long-term Bti programs is the control of *Aedes vexans* in the Upper Rhine Valley, Germany, which has been in operation since the 1980s [15], along with programs carried out in other regions [6, 17, 18, 53].

In our investigation, the in vivo susceptibility of RecBti larvae to individual Cry11Aa and Cry4Ba protoxins has also been evaluated, and F_50_ larvae revealed a threefold increase in resistance ratios (RRs). A comparison with RecBti susceptibility data available showed that variations of such magnitude were also observed at earlier time points, including generations F_10_ and F_30_ [23, 40], with no consistent trend of increasing resistance over time. To better address this aspect, transcript levels of genes encoding APN, ALP, and a CAD, previously reported as potential Cry11Aa receptors [45–49], were found to be higher than those observed in control strains. Given that Cry11Aa is the most toxic individual component of the Bti crystal, the continued expression of its receptors in RecBti larvae corroborates the observed in vivo susceptibility. The expression of such midgut receptor genes in larvae is needed for the action of Bti Cry toxins. In other studies, reduced transcription of these receptors has been associated with resistance in strains selected with individual toxins or the complete Bti crystal [21, 47, 54]. The RecBti larvae, chronically exposed to Bti and susceptible to this agent, showed no differential expression of these receptor genes in the RT-qPCR search nor in the transcriptomic profile of RecBti midguts by ribonucleic acid sequencing (RNAseq) [40]. Nevertheless, this aspect should be seen with caution, as recent studies showed that the knockdown of such proteins did not impact the in vivo susceptibility of *Ae. aegypti* [55, 56], suggesting a more complex scenario of molecules responsible for this phenotype. In addition, it has been also demonstrated that the role of midgut receptors during different larval stages might display changes [57], which makes it difficult to assign the specific role of these molecules for the in vivo mortality achieved. Therefore, the increased relative quantification (Rq) of the three binding sites detected in RecBti larvae in this study remains to be better investigated, as a similar quantification or RecBti and RecL could be expected. In the transcriptome profile, other potential resistance mechanisms associated with Bti toxicity, such as impaired protoxin activation or upregulation of detoxifying enzymes, were not detected in the RecBti strain [40]. Together, these findings from the assessment of this strain, prolonged and intensively exposed to Bti, reinforce that Bti has a low potential for resistance selection.

In addition to monitoring the susceptibility of the RecBti strain to Bti, we investigated other biological traits that could be influenced by long-term larvicide exposure. This inquiry was motivated by the transcriptomic data from RecBti larvae at generation F_35_, which revealed genes associated with vitellogenesis, coding for the vitellogenin carboxypeptidase precursor and cathepsin B, among the top five most upregulated [40]. RecBti eggs demonstrated higher hatching rates after prolonged quiescence, suggesting enhanced egg viability, while adults showed increased longevity. The significantly higher lipid reserves found for RecBti larvae and adults compared with the control strain are also a trait that may confer an advantage under environmental stress and influence flavivirus replication [58]. A previous assessment of RecBti life traits in terms of fertility, fecundity, pre-imaginal period, pupae weight, and hematophagy capacity did not reveal differences between the strains. However, the changes in the parameters seen in the present study, combined with previously reported increased susceptibility of RecBti females to Zika virus [41], suggest that the prolonged Bti exposure might provoke alterations in the ability of this species to be in contact with humans and transmit pathogens. The influence of exposure to larvicides and other effects on mosquitoes’ abilities to transmit pathogens has been raised by different studies [59–61]. These findings highlight the importance of assessing not only resistance onset but also having a broader view of the biological traits of mosquitoes subjected to continuous larvicide exposure. There is a growing recognition of the effects of insecticides on mosquito biology, including those mediated by the bacterial microbiota, that can play essential roles in mosquito metabolism, physiology, and development [62–64]. Although not investigated in this study, it is plausible that mosquito exposure to microbial larvicides may influence their microbiota, with effects on development and vector competence [65, 66].

Among the desirable attributes for sustainable larvicide use, the absence of reported resistance to Bti stands out, especially considering the widespread resistance reported for other larvicide classes, including conventional chemical insecticides [67, 68], insect growth regulators [69], and spinosyns [70, 71]. This feature, positions Bti as a strategic tool for integration with other control agents. Long-lasting larvicides combining Bti and *Lysinibacillus sphaericus* crystals have been developed to target a wide range of mosquito species and ecological niches [72, 73], while also mitigating resistance issues associated with *L. sphaericus*-only products [74]. Notably, Cyt1Aa, a key toxin in the Bti crystal, can synergize with the action of the binary toxin from *L. sphaericus*, thereby enhancing efficacy and reducing the risk of resistance [75]. In a comparative study, *Culex quinquefasciatus* strains selected with Spinosad alone developed high resistance (RR ~50-fold), while no resistance was observed in a strain selected with a combination of Spinosad–Bti [70]. These findings further support the central role of Bti insecticidal toxins in resistance management strategies.

## Conclusions

Our data demonstrated that Bti-based larvicides have a low risk of resistance selection, providing robust evidence of Sustained susceptibility after 50 generations under prolonged selection pressure, and supports the notion that Bti remains unique in terms of low risk of resistance onset. These findings show the outstanding status of Bti as a larvicide with no documented field resistance to its insecticidal crystal and its importance in managing resistance to other compounds. Beyond resistance, our results suggest that long-term Bti exposure may influence biological traits related to survival and reproduction, underscoring the importance of evaluating broader biological effects associated with microbial larvicide use.

## Supplementary Information

**Additional file 1: Table S1.** Primers used to amplify *Aedes aegypti* genes encoding Cry toxin receptors.

**Additional file 2: Table S2.** Exposure of *Aedes aegypti* third instar larvae from the RecBti strain to *Bacillus thuringiensis *svar.* israelensis* under laboratory conditions.

**Additional file 3: Table S3.** Dataset of the dose response bioassays to evaluate the toxicity of the *Bacillus thuringiensis* svar. *israelensis* (Bti) larvicide and its Cry11Aa and Cry4Ba protoxins to third instar larvae of *Aedes aegypti* from the Rockefeller (Rocke reference) strain and the RecBti strain exposed to Bti during fifty generations. Larvae from F_40_ and F_50_ generations were assessed.

**Additional file 4: Table S4.** Dataset of the RT-qPCR assays for the relative quantification of the cry receptors transcripts in pool of ten third instar larvae of *Aedes aegypti* from RecBti and RecL strain. Ct. Cycle threshold. Rq. Relative quantification. A. Average. SD. Standard deviation. R. Reference sample.

**Additional file 5: Table S5.** Dataset of lipids and reducing sugars quantification in third instar larvae and adults of *Aedes aegypti* from RecBti and RecL strains. Lipids in pools of 10 larvae and in individual females. Reducing sugars in pools of 20 larvae and individual females. Absorbance at 540 nm (Abs). Test sample incubated with amyloglucosidase (T). Negative control sample incubated without enzyme (C). Amount of reducing sugars (S).

**Additional file 6: Table S6.** Dataset of egg viability from an *Aedes aegypti* strain exposed to *Bacillus thuringiensis* svar. *israelensis* for fifty generations (RecBti) compared with a reference strain (RecL). Egg samples were stored between 3 and 180 days after oviposition. Replicates (R) were samples (n > 80 eggs) submerged in water with grass infusion (6 g/L), and first instar larvae were recorded daily until 96 h.

**Additional file 7: Table S7.** Dataset of the longevity assay of *Aedes aegypti* from RecBti and RecL strain, adults stratified by sex. Replicates were groups of 50 adults (1:1 sex ratio) kept with 10% sucrose and water ad libitum. M. Male. F. Female. Two independent assays (1, 2) were done.

## Data Availability

Data supporting the main conclusions of this study are included in the manuscript.

## Abbreviations

ALP: Alkaline phosphatase
APN: Aminopeptidase N
Bti: *Bacillus* *thuringiensis* svar. *israelensis*
CAD: Cadherin
CI: Confidence interval
Cry: Crystal
Ct: Cycle threshold
Cyt: Cytolytic toxin
Fiocruz: Fundação Oswaldo Cruz
GLU: Glucosidase
IAM: Instituto Aggeu Magalhães
IPS82: Bti reference technical powder
ITU: International toxic units
LC: Lethal concentration
P: Parental generation
RecBti: *Aedes aegypti* strain selected to Bti
Rocke: *Aedes aegypti* Rockefeller international reference strain
RecL: *Aedes aegypti* local reference strain
RT-qPCR: Real time quantitative polymerase chain reaction
RR: Resistance ratio
RT: Room temperature
WHO: World Health Organization
