# Supplementary material for: Aedes aegypti strain selected with Bacillus thuringiensis svar. israelensis larvicide for 50 generations remains susceptible and exhibited increased fitness
Source: Parasit Vectors. 2025 Oct 7;18:400. doi: 10.1186/s13071-025-07037-x (PMC12506322; doi:10.1186/s13071-025-07037-x)
Supplement: Supplementary file 1 — Additional file 1: Table S1. Primers used to amplify Aedes aegypti genes encoding Cry toxin receptors. [file 13071_2025_7037_MOESM1_ESM.pdf]

Additional file 1: Table S1. Primers used to amplify *Aedes aegypti* genes encoding Cry toxin receptors.

Gene[a]	Primers 5'-3'	Amplicon[b]
APN (AAEL012778)	F GAGTACTTATCGCACCAAAACCAAC<br>R GAAAAGCTCCCAATATCCCACCTC	187
ALP (AAEL015070)	F GGAAGCGCACAAGGAGAACAA<br>R ATGAAGGCCCCGTAGCGTAAG	246
CAD (AAEL007478)	F CGAGACGCTGTTCCTCACG<br>R CGAACCGATCTACTTCCAACG	149
18S	F CGCGGTAATTCCAGCTCCACTA<br>R GCATCAAGCGCCACCATATAGG	159

[a] APN aminopeptidase. ALP-alkaline phosphatase. CAD-cadherin.

[b] Size in basepairs
