# Supplementary material for: Aedes aegypti strain selected with Bacillus thuringiensis svar. israelensis larvicide for 50 generations remains susceptible and exhibited increased fitness
Source: Parasit Vectors. 2025 Oct 7;18:400. doi: 10.1186/s13071-025-07037-x (PMC12506322; doi:10.1186/s13071-025-07037-x)
Supplement: Supplementary file 2 — Additional file 2: Table S2. Exposure of Aedes aegypti third instar larvae from the RecBti strain to Bacillus thuringiensis svar. israelensis under laboratory conditions. [file 13071_2025_7037_MOESM2_ESM.pdf]

**Additional file 2: Table S2.** Exposure of *Aedes aegypti* third instar larvae from the RecBti strain to *Bacillus thuringiensis* svar. *israelensis* under laboratory conditions.

| Generation | No. larvae[a] | Mortality 24 h (%) | Mortality[b] (%) | No. adults |
|---|---|---|---|---|
| Parental[c] | 11.600 | ND[d] | 74 | 3000 |
| $F_1$ | 8350 | ND | 69 | 2573 |
| $F_2$ | 5700 | ND | 79 | 1209 |
| $F_3$ | 12.000 | ND | 77 | 2702 |
| $F_4$ | 7800 | ND | 63 | 3082 |
| $F_5$ | 8100 | 58 | 69 | 2531 |
| $F_6$ | 17.700 | 75 | 85 | 2590 |
| $F_7$ | 11.100 | 50 | 76 | 2682 |
| $F_8$ | 8700 | 53 | 69 | 2691 |
| $F_9$ | 10.200 | 57 | 72 | 2506 |
| $F_{10}$ | 14.700 | 80 | 91 | 1322 |
| $F_{11}$ | 6400 | 59 | 63 | 2530 |
| $F_{12}$ | 10.500 | 59 | 84 | 3440 |
| $F_{13}$ | 12.900 | 65 | 88 | 1557 |
| $F_{14}$ | 11.200 | 64 | 85 | 1717 |
| $F_{15}$ | 21.600 | 66 | 76 | 5182 |
| $F_{16}$ | 11.600 | 60 | 69 | 3570 |
| $F_{17}$ | 9600 | 65 | 75 | 2412 |
| $F_{18}$ | 9200 | 59 | 71 | 2617 |
| $F_{19}$ | 6400 | 65 | 74 | 1828 |
| $F_{20}$ | 9600 | 57 | 68 | 3023 |
| $F_{21}$ | 7200 | 66 | 74 | 1812 |
| $F_{22}$ | 6800 | 59 | 73 | 1836 |
| $F_{23}$ | 7200 | 58 | 72 | 1982 |
| $F_{24}$ | 6800 | 56 | 75 | 1099 |
| $F_{25}$ | 6000 | 51 | 79 | 1252 |
| $F_{26}$ | 6000 | 52 | 76 | 1651 |
| $F_{27}$ | 6400 | 47 | 66 | 2162 |
| $F_{28}$ | 6800 | 64 | 79 | 1430 |
| $F_{29}$ | 8000 | 45 | 66 | 2723 |
| $F_{30}$ | 6400 | 45 | 65 | 1938 |
| $F_{31}$ | 6000 | 49 | 75 | 1.936 |
| $F_{32}$ | 6000 | 57 | 75 | 1.468 |
| $F_{33}$ | 6800 | 51 | 74 | 1.668 |
| $F_{34}$ | 7200 | 45 | 72 | 2.102 |
| $F_{35}$ | 6400 | 46 | 61 | 2.476 |
| $F_{36}$ | 6000 | 41 | 65 | 2660 |
| $F_{37}$ | 6000 | 42 | 60 | 1815 |
| $F_{38}$ | 6000 | 55 | 70 | 2.692 |
| $F_{39}$ | 7200 | 53 | 65 | 2.538 |
| $F_{40}$ | 6800 | 58 | 60 | 2.738 |
| $F_{41}$ | 4000 | 44 | 85 | 613 |
| $F_{42}$ | 6800 | 55 | 56 | 2.976 |
| $F_{43}$ | 4400 | 53 | 56 | 1.944 |
| $F_{44}$ | 6000 | 53 | 55 | 2.716 |
| $F_{45}$ | 6000 | 39 | 68 | 1942 |

| | | | | |
|---|---|---|---|---|
| $F_{46}$ | 6500 | 57 | 79 | 1393 |
| $F_{47}$ | 9750 | 60 | 63 | 2619 |
| $F_{48}$ | 14.500 | 56 | 83 | 1198 |
| $F_{49}$ | 11.080 | 61 | 86 | 1570 |
| $F_{50}$ | 11.200 | 60 | 83 | 1890 |
| **Mean** | 8572.1 | 55.9 | 72.4 | 2227.5 |
| **SD** | 3384.8 | 8.5 | 8.7 | 773.9 |

[a] Third instar larvae from the RecBti colony treated with VectoBac® WG (0.5 mg/L).

[b] Final mortality based on the number of adult survivors.

[c] Parental generation established with eggs collected in 40 neighborhoods of Recife city

[d] Not determined
