# Supplementary material for: Aedes aegypti strain selected with Bacillus thuringiensis svar. israelensis larvicide for 50 generations remains susceptible and exhibited increased fitness
Source: Parasit Vectors. 2025 Oct 7;18:400. doi: 10.1186/s13071-025-07037-x (PMC12506322; doi:10.1186/s13071-025-07037-x)
Supplement: Supplementary file 3 — Additional file 3: Table S3. Dataset of the dose response bioassays to evaluate the toxicity of the Bacillus thuringiensis svar. israelensis (Bti) larvicide and its Cry11Aa and Cry4Ba protoxins to third instar larvae of Aedes aegypti from the Rockefeller (Rocke reference) strain and the RecBti strain exposed to Bti during fifty generations. Larvae from F40 and F50 generations were assessed. [file 13071_2025_7037_MOESM3_ESM.pdf]

Additional file 3: Table S3. Dataset of the dose response bioassays to evaluate the toxicity of the *Bacillus thuringiensis* svar. *israelensis* (Bti) larvicide and its Cry11Aa and Cry4Ba protoxins to third instar larvae of *Aedes aegypti* from the Rockefeller (Rocke reference) strain and the RecBti strain exposed to Bti during fifty generations. Larvae from F_{40} and F_{50} generations were assessed.

**F_{40}**	**Rocke**				**RecBti**			
	**No**	**LC_{50} (CI 95%) [a]**	**LC_{90} (CI 95%) [a]**	**Qui-square**	**No**	**LC_{50} (CI 95%) [a]**	**LC_{90} (CI 95%) [a]**	**Qui-square[b]**
Bti	420	0.015 (0.014-0.017)	0.033 (0.029-0.038)	1.96	300	0.021 (0.018-0.023)	0.045 (0.037-0.059)	0.48
	300	0.014 (0.012-0.016)	0.033 (0.027-0.043)	0.71	420	0.016 (0.014-0.019)	0.044 (0.037-0.055)	4.51
	480	0.014 (0.012-0.016)	0.033 (0.028- 0.041)	3.87	360	0.014 (0.012-0.016)	0.029 (0.025-0.035)	3.90
					300	0.014 (0.012-0.017)	0.029 (0.025-0.036)	1.92
Total	1200	0.014 (0.013-0.016)	0.033 (0.028- 0.041)		1380	0.016 (0.014 - 0.019)	0.036 (0.031- 0.046)	
Cry11Aa	480	1.202 (0.958-1.549)	ND	4.23	480	1.329 (1.010-1.838)	ND	1.19
	480	0.956 (0.771-1.200)		2.94	480	1.062 (0.833-1.383)		2.15
	420	1.063 (0.833-1.378)		5.44	420	1.307 (0.938-1.945)		1.83
	480	0.981 (0.649-1.560)		8.79				
	1860	1.051 (0.803-1.422)			1.380	1.232 (0.927-1.722)		
Cry4Ba	480	0.835 (0.634 - 1.104)	ND	2.59	360	1.286 (0.812-2.273)	ND	1.37
	480	0.927 (0.534 - 1.585)		1.27	420	1.054 (0.767-1.495)		1.15
	480	0.959 (0.699 - 1.343)		1.97	420	1.194 (0.846-1.778)		2.82
Total	1.440	0.907 (0.622 - 1.344)			1.200	1.178 (0.808-1.849)		
**F_{50}**	**Rocke**				**RecBti**			
	**No**	**LC_{50} (CI 95%)**	**LC_{90} (CI 95%)**		**No**	**LC_{50} (CI 95%)**	**LC_{90} (CI 95%)**	**Qui-square**
Bti	360	0.009 (0.007-0.010)	0.023 (0.020-0.030)	4.99	420	0.016 (0.013-0.020)	0.031 (0.026-0.039)	5.672
	300	0.010 (0.006-0.013)	0.023 (0.017-0.041)	6.14	420	0.013 (0.011-0.014)	0.032 (0.028-0.040)	2.22

	360	0.010 (0.009-0.011)	0.022 (0.019-0.027)	2.98	360	0.015 (0.014-0.017)	0.028(0.026-0.032)	5.70
					360	0.018 (0.016-0.020)	0.032 (0.029-0.036)	4.53
Total	1020	0.001 (0.007-0.011)	0.023 (0.019-0.033)		1560	0.016 (0.014-0.018)	0.031 (0.027-0.037)	
Cry11Aa	420	0.326 (0.246-0.422)	ND	1.48	480	0.508 (0.371-0.674)	ND	0.94
	420	0.295 (0.214-0.389)		2.12	360	1.608 (1.135-2.156)		1.87
	420	0.283 (0.150-0.468)		1.55	360	0.601 (0.346-0.928)		1.17
	360	0.325 (0.215-0.453)		0.98				
Total	1620	0.307 (0.206-0.433)			1200	0.906 (0.617-1.253)		
Cry4Ba	360	0.656 (0.432-1.028)	ND	1.46	360	1.602 (0.909-2.667)	ND	0.38
	360	0.370 (0.273-0.486)		3.62	360	1.595 (0.910-3.716)		0.78
	360	0.633 (0.429-0.854)		4.08				
Total	1080	0.553 (0.378-0.789)			720	1.599 (0.910-3.192)		

[a] Lethal Concentration (mg/L) for 50% to 90% of larvae after 48h of exposure with a 95% of confidence interval.
[b] Heterogeneity data obtained from the ratio between chi-square.
