# Supplementary material for: Aedes aegypti strain selected with Bacillus thuringiensis svar. israelensis larvicide for 50 generations remains susceptible and exhibited increased fitness
Source: Parasit Vectors. 2025 Oct 7;18:400. doi: 10.1186/s13071-025-07037-x (PMC12506322; doi:10.1186/s13071-025-07037-x)
Supplement: Supplementary file 4 — Additional file 4: Table S4. Dataset of the RT-qPCR assays for the relative quantification of the cry receptors transcripts in pool of ten third instar larvae of Aedes aegypti from RecBti and RecL strain. Ct. Cycle threshold. Rq. Relative quantification. A. Average. SD. Standard deviation. R. Reference sample. [file 13071_2025_7037_MOESM4_ESM.pdf]

Additional file 4: Table S4. Dataset of the RT-qPCR assays for the relative quantification of the Cry receptors transcripts in pool of ten third instar larvae of *Aedes aegypti* from RecBti and RecL strains. Ct. Cycle threshold. Rq. Relative quantification. A. Average. SD. Standard deviation. R. Reference sample.

**RecL**						**RecBti**				
**Aminopeptidase**										
**N**	**CT 18S**	**Ct CAD**	**ΔCt**	**ΔΔCt**	**Rq**	**CT 18S**	**Ct CAD**	**ΔCt**	**ΔΔCt**	**Rq**
R	27,11	24,25	-2,86	0,00	1,00	27,11	24,25	-2,86	0,00	1,00
1	27,56	29,82	2,26	0,18	0,88	26,60	24,37	-2,23	-4,31	19,81
2	27,22	27,27	0,05	-2,03	4,08	26,57	25,87	-0,70	-2,78	6,88
3	28,61	28,40	-0,21	-2,29	4,87	26,85	21,81	-5,04	-7,12	139,49
4	29,71	29,90	0,19	-1,89	3,7	24,62	22,13	-2,49	-4,57	23,69
5	26,88	27,23	0,35	-1,73	3,32	28,42	23,00	-5,41	-7,49	180,143
6	26,74	27,39	0,65	-1,43	2,69	29,21	27,60	-1,62	-3,70	12,987
7	27,48	27,14	-0,33	-2,41	5,32	26,47	25,99	-0,48	-2,56	5,90
8	26,26	29,85	3,59	1,51	0,35	27,50	24,16	-3,34	-5,42	42,78
9	29,16	26,34	-2,82	-4,90	29,82	26,76	22,71	-4,05	-6,13	70,18
10	29,58	27,57	-2,02	-4,10	17,09	25,33	20,72	-4,62	-6,70	103,61
11	29,84	28,62	-1,21	-3,29	9,81	25,33	20,72	-4,61	-6,69	103,32
12	28,61	27,25	-1,36	-3,44	10,85	26,94	21,36	-5,59	-7,67	202,95
13	29,77	27,36	-2,41	-4,49	22,49	27,72	25,47	-2,25	-4,33	20,11
14	28,85	26,98	-1,87	-3,95	15,44	27,23	21,15	-6,09	-8,17	287,62
15	28,66	28,89	0,23	-1,85	3,61	26,14	22,73	-3,41	-5,49	45,07
16	28,84	27,09	-1,75	-3,83	14,21	26,99	21,66	-5,33	-7,41	169,72
17	29,49	26,73	-2,76	-4,84	28,58	29,16	23,43	-5,73	-7,81	224,72
18	27,93	25,94	-1,99	-4,07	16,77	27,47	21,41	-6,06	-8,14	281,70
19	27,94	26,03	-1,91	-3,99	15,88	26,35	23,70	-2,65	-4,73	26,56
**A**	28,61	27,27	-1,21	-3,29	**11,04**	26,853	22,728	-4,053	-6,133	**103,54**
**SD**	1,09	1,39	1,68	1,73	**8,91**	1,13	1,90	1,73	2,14	**93,08**
**Alkaline phosphatase**										
**N**	**CT 18S**	**Ct CAD**	**ΔCt**	**ΔΔCt**	**Rq**	**CT 18S**	**Ct CAD**	**ΔCt**	**ΔΔCt**	**Rq**

R	28,22	32,21	3,99	0,00	1,00	28,22	32,21	3,99	0,00	1,00
1	28,77	32,54	3,77	-0,22	1,16	27,58	31,63	4,05	0,06	0,96
2	29,28	32,61	3,33	-0,66	1,58	26,88	29,85	2,97	-1,02	2,03
3	27,93	32,37	4,44	0,45	0,73	26,97	27,49	0,52	-3,47	11,06
4	28,50	34,77	6,28	2,29	0,21	25,66	33,64	7,98	3,99	0,06
5	27,99	30,76	2,78	-1,22	2,32	25,20	33,62	8,43	4,44	0,05
6	28,06	32,37	4,31	0,32	0,80	27,41	34,10	6,69	2,70	0,15
7	27,73	32,12	4,39	0,40	0,76	28,03	29,60	1,57	-2,42	5,34
8	30,64	32,19	1,54	-2,45	5,46	28,27	27,84	-0,43	-4,42	21,33
9	30,24	31,77	1,53	-2,46	5,51	26,99	25,87	-1,12	-5,11	34,44
10	31,43	33,89	2,46	-1,53	2,89	29,16	27,29	-1,87	-5,86	58,08
11	30,48	31,57	1,09	-2,90	7,47	27,47	25,04	-2,43	-6,42	85,87
12	30,49	31,34	0,85	-3,14	8,82	26,35	25,18	-1,18	-5,17	35,90
13	30,21	31,93	1,71	-2,28	4,84	29,79	25,40	-4,39	-8,38	333,61
14	30,56	32,54	1,99	-2,00	4,01	29,71	26,80	-2,91	-6,90	119,43
15	30,22	31,93	1,71	-2,28	4,85	29,13	26,95	-2,18	-6,17	72,05
16	28,66	31,64	2,98	-1,01	2,02	29,03	26,25	-2,77	-6,76	108,68
17	28,84	31,77	2,93	-1,06	2,08					
18	29,49	31,95	2,46	-1,53	2,89					
19	27,93	32,09	4,16	0,17	0,89					
20	27,94	32,18	4,24	0,25	0,84					
**A**	29,279	32,09	2,78	-1,22	**3.01**	27,47	27,29	-1,12	-5,11	**55.56**
**SD**	1,14	0,84	1,37	1,37	**2,38**	1,34	3,06	3,95	3,95	**81,77**

Cadherin

**N**	**CT 18S**	**Ct CAD**	**ΔCt**	**ΔΔCt**	**Rq**	**CT 18S**	**Ct CAD**	**ΔCt**	**ΔΔCt**	**Rq**
R	27,11	24,25	-2,86	0,00	1,00	27,11	24,25	-2,86	0,00	1,00
1	26,94	28,51	1,57	0,65	0,64	24,68	23,77	-0,91	-1,83	3,55
2	26,46	27,38	0,92	0,00	1,00	26,35	24,77	-1,59	-2,51	5,68
3	26,86	27,41	0,55	-0,37	1,29	26,94	23,67	-3,27	-4,19	18,30
4	26,10	26,28	0,18	-0,74	1,68	24,78	25,20	0,42	-0,50	1,42
5	25,91	26,71	0,80	-0,12	1,09	24,84	24,12	-0,73	-1,65	3,13

6	26,11	28,22	2,11	1,19	0,44	25,52	25,58	0,07	-0,85	1,81
7	27,26	27,09	-0,17	-1,09	2,13	25,82	24,78	-0,06	-0,98	1,97
8	25,57	27,40	1,83	0,91	0,53	26,34	27,33	1,81	0,89	0,54
9	26,80	28,29	1,50	0,58	0,67	26,64	26,78	0,96	0,04	0,98
10	28,66	30,83	2,17	1,25	0,42	26,99	24,88	-2,11	-3,03	8,17
11	28,8	28,52	-0,32	-1,24	2,36	29,16	27,06	-2,10	-3,02	8,13
12	29,49	30,70	1,21	0,29	0,82	27,47	25,33	-2,14	-3,06	8,36
13	27,93	30,14	2,21	1,29	0,41	26,35	25,52	-0,83	-1,75	3,36
14	27,94	29,63	1,69	0,77	0,58	26,11	24,85	-1,26	-2,18	4,52
15	26,22	27,36	1,14	0,22	0,86	25,83	23,38	-2,46	-3,38	10,40
16	25,45	27,76	2,31	1,39	0,38	27,45	25,08	-2,37	-3,29	9,79
17	26,82	28,94	2,12	1,20	0,43	26,12	22,80	-3,32	-4,24	18,92
18						24,55	23,10	-1,45	-2,37	5,17
**A**	26,82	28,22	1,50	0,58	**0,92**	26,23	24,86	-1,35	-2,27	**6,34**
**SD**	1,15	1,32	0,82	0,82	**0,59**	1,13	1,26	1,38	1,38	**5,26**

Statistical data analysis

**Normality Test**			**Unpaired t test with Welch's correction**	
**APN**				
**Shapiro-W**	**RecL**	**RecBti**	P value	<0,0001
W	0,5586	0,8706	Exact or ap	Exact
P value	<0,0001	0,0148	Significantl	Yes
Passed nor	No	No	One- or twc	Two-tailed
P value sur	****	*	Mann-Whit	80
**ALP**				
**Shapiro-Wilk test**			P value	0,0156
W	0,853	0,7389	Exact or ap	Exact
P value	0,0025	0,0003	Significantl	Yes
Passed nor	No	No	One- or twc	Two-tailed

P value sur	**	***		Mann-Whit	95

CAD

**Shapiro-Wilk test**				P value	<0,0001
W	0,3378	0,8561		Exact or ap	Exact
P value	<0,0001	0,0106		Significantl	Yes
Passed nor	No	No		One- or twc	Two-tailed
P value sur	****	*		Mann-Whit	42
