# Supplementary material for: Aedes aegypti strain selected with Bacillus thuringiensis svar. israelensis larvicide for 50 generations remains susceptible and exhibited increased fitness
Source: Parasit Vectors. 2025 Oct 7;18:400. doi: 10.1186/s13071-025-07037-x (PMC12506322; doi:10.1186/s13071-025-07037-x)
Supplement: Supplementary file 5 — Additional file 5: Table S5. Dataset of lipids and reducing sugars quantification in third instar larvae and adults of Aedes aegypti from RecBti and RecL strains. Lipids in pools of 10 larvae and in individual females. Reducing sugars in pools of 20 larvae and individual females. Absorbance at 540 nm (Abs). Test sample incubated with amyloglucosidase (T). Negative control sample incubated without enzyme (C). Amount of reducing sugars (S). [file 13071_2025_7037_MOESM5_ESM.pdf]

Additional file 5: Table S5. Dataset of thequantification of lipids and reducing sugars in third instar larvae and adults of *Aedes aegypti* from RecBti and RecL strains. Lipids in pools of 10 larvae and in individual females. Reducing sugars in pools of 20 larvae and individual females. Absorbance at 540 nm (Abs). Test sample incubated with amyloglucosidase (T). Negative control sample incubated without enzyme (C). Amount of reducing sugars (S).

LIPID

	LARVAE				ADULTS			
	ABS		Lipid (ug)		ABS		Lipid (ug)	
N	RecL	RecBti	RecL	RecBti	RecL	RecBti	RecL	RecBti
1	0,544	1,137	82,18	163,41	0,351	0,653	55,74	97,11
2	0,563	0,83	84,78	131,28	0,375	0,551	59,03	83,14
3	0,601	0,942	89,99	148,00	0,324	0,427	52,04	66,15
4	0,49	0,898	74,78	141,43	0,594	0,615	89,03	91,90
5	0,388	0,912	60,81	143,52	0,458	0,406	70,40	63,27
6	0,664	0,979	98,62	153,52	0,458	0,731	70,40	107,79
7	0,448	1,318	69,03	204,12	0,441	0,422	68,07	65,47
8	0,631	1,137	94,10	177,10	0,406	0,628	63,27	93,68
9	0,626	0,915	93,41	143,97	0,49	0,557	74,78	83,96
10	0,527	0,922	79,85	145,01	0,437	0,497	67,52	75,74
11	0,592	1,089	88,75	169,94	0,475	0,574	72,73	86,29
12	0,645	1,24	96,01	192,48	0,409	0,572	63,68	86,01
13	0,671	0,941	99,58	147,85	0,448	0,654	69,03	97,25
14	0,739	1,199	108,89	186,36	0,378	0,62	59,44	92,59
15	0,637	1,101	94,92	171,73	0,4	0,582	62,45	87,38
16	0,779	1,217	114,37	189,04	0,379	0,546	59,58	82,45
17	0,626	0,869	93,41	137,10	0,447	0,479	68,89	73,27
18	0,765	1,19	112,45	185,01	0,444	0,592	68,48	88,75
19	0,579	1,245	86,97	193,22	0,42	0,472	65,19	72,32
20	0,782	1,215	114,78	188,75	0,4	0,417	62,45	64,78
**A**	**0,61**	**1,06**	**91,88**	**165,64**	**0,43**	**0,55**	**66,11**	**82,97**
**SD**	**0,10**	**0,15**	**14,12**	**22,11**	**0,06**	**0,09**	**7,72**	**12,14**

REDUCING SUGARS

LARVAE RecL	ADULTS RecL

N	ABS Sample	ABS Blank	Curve value Sample	Curve value Blank	Reducing Sugar	mg Total	ug Total	ABS Sample	ABS Blank	Curve value Sample	Curve value Blank	Reducing Sugar	mg Total	ug Total
1	0,494	0,242	0,33	0,10	0,23	**0,27**	**274,51**	0,708	0,522	0,53	0,36	**0,17**	**0,20**	**202,61**
2	0,727	0,515	0,54	0,35	0,19	**0,23**	**230,94**	0,639	0,421	0,46	0,27	**0,20**	**0,24**	**237,47**
3	0,838	0,572	0,65	0,40	0,24	**0,29**	**289,76**	0,692	0,503	0,51	0,34	**0,17**	**0,21**	**205,88**
4	0,777	0,528	0,59	0,36	0,23	**0,27**	**271,24**	0,715	0,527	0,53	0,36	**0,17**	**0,20**	**204,79**
5	0,654	0,453	0,48	0,30	0,18	**0,22**	**218,95**	0,578	0,369	0,41	0,22	**0,19**	**0,23**	**227,67**
6	0,662	0,322	0,49	0,18	0,31	**0,37**	**370,37**	0,746	0,514	0,56	0,35	**0,21**	**0,25**	**252,72**
7	0,701	0,311	0,52	0,17	0,35	**0,42**	**424,84**	0,589	0,401	0,42	0,25	**0,17**	**0,20**	**204,79**
8	0,802	0,578	0,61	0,41	0,20	**0,24**	**244,01**	0,693	0,512	0,51	0,35	**0,16**	**0,20**	**197,17**
9	0,811	0,523	0,62	0,36	0,26	**0,31**	**313,73**	0,708	0,522	0,53	0,36	**0,17**	**0,20**	**202,61**
10	0,784	0,569	0,60	0,40	0,20	**0,23**	**234,20**	0,639	0,421	0,46	0,27	**0,20**	**0,24**	**237,47**
11	0,634	0,452	0,46	0,29	0,17	**0,20**	**198,26**	0,692	0,503	0,51	0,34	**0,17**	**0,21**	**205,88**
12	0,698	0,487	0,52	0,33	0,19	**0,23**	**229,85**	0,715	0,527	0,53	0,36	**0,17**	**0,20**	**204,79**
13	0,726	0,503	0,54	0,34	0,20	**0,24**	**242,92**	0,578	0,369	0,41	0,22	**0,19**	**0,23**	**227,67**
14	0,793	0,566	0,60	0,40	0,21	**0,25**	**247,28**	0,746	0,514	0,56	0,35	**0,21**	**0,25**	**252,72**
15	0,648	0,475	0,47	0,32	0,16	**0,19**	**188,45**	0,589	0,401	0,42	0,25	**0,17**	**0,20**	**204,79**
16	0,762	0,527	0,58	0,36	0,21	**0,26**	**255,99**	0,693	0,512	0,51	0,35	**0,16**	**0,20**	**197,17**
17	0,687	0,503	0,51	0,34	0,17	**0,20**	**200,44**	0,642	0,471	0,47	0,31	**0,16**	**0,19**	**186,27**
18	0,715	0,486	0,53	0,33	0,21	**0,25**	**249,46**	0,701	0,514	0,52	0,35	**0,17**	**0,20**	**203,70**
19								0,608	0,422	0,44	0,27	**0,17**	**0,20**	**202,61**
20								0,7	0,497	0,52	0,34	**0,18**	**0,22**	**221,13**
21								0,709	0,502	0,53	0,34	**0,19**	**0,23**	**225,49**
22								0,623	0,476	0,45	0,32	**0,13**	**0,16**	**160,13**
**A**							**260,29**							**212,07**
**SD**							**58,16**							**0,00**

	LARVAE RecBti							ADULTS RecBti						
N	ABS Sample	ABS Blank	Curve value Sample	Curve value Blank	Reducing Sugar	mg Total	ug Total	ABS Sample	ABS Blank	Curve value Sample	Curve value Blank	Reducing Sugar	mg Total	ug Total
1	0,759	0,553	0,57	0,39	**0,19**	**0,22**	**224,40**	0,423	0,229	0,27	0,09	**0,18**	**0,21**	**211,33**
2	0,851	0,683	0,66	0,50	**0,15**	**0,18**	**183,01**	0,408	0,217	0,25	0,08	**0,17**	**0,21**	**208,06**
3	0,589	0,41	0,42	0,26	**0,16**	**0,19**	**194,99**	0,572	0,399	0,40	0,25	**0,16**	**0,19**	**188,45**
4	0,652	0,441	0,48	0,28	**0,19**	**0,23**	**229,85**	0,579	0,432	0,41	0,28	**0,13**	**0,16**	**160,13**
5	0,55	0,37	0,38	0,22	**0,16**	**0,20**	**196,08**	0,588	0,372	0,42	0,22	**0,20**	**0,24**	**235,29**
6	0,559	0,397	0,39	0,24	**0,15**	**0,18**	**176,47**	0,621	0,402	0,45	0,25	**0,20**	**0,24**	**238,56**

7	0,618	0,345	0,45	0,20	**0,25**	**0,30**	**297,39**	0,582	0,405	0,41	0,25	**0,16**	**0,19**	**192,81**
8	0,546	0,318	0,38	0,17	**0,21**	**0,25**	**248,37**	0,609	0,388	0,44	0,24	**0,20**	**0,24**	**240,74**
9	0,396	0,234	0,24	0,10	**0,15**	**0,18**	**176,47**	0,486	0,229	0,33	0,09	**0,23**	**0,28**	**279,96**
10	0,445	0,204	0,29	0,07	**0,22**	**0,26**	**262,53**	0,643	0,465	0,47	0,31	**0,16**	**0,19**	**193,90**
11	0,453	0,263	0,30	0,12	**0,17**	**0,21**	**206,97**	0,549	0,326	0,38	0,18	**0,20**	**0,24**	**242,92**
12	0,577	0,264	0,41	0,12	**0,28**	**0,34**	**340,96**	0,621	0,419	0,45	0,26	**0,18**	**0,22**	**220,04**
13	0,577	0,308	0,41	0,16	**0,24**	**0,29**	**293,03**	0,483	0,235	0,32	0,10	**0,23**	**0,27**	**270,15**
14	0,537	0,311	0,37	0,17	**0,21**	**0,25**	**246,19**	0,645	0,397	0,47	0,24	**0,23**	**0,27**	**270,15**
15	0,507	0,269	0,34	0,13	**0,22**	**0,26**	**259,26**	0,627	0,452	0,45	0,29	**0,16**	**0,19**	**190,63**
16	0,462	0,28	0,30	0,14	**0,17**	**0,20**	**198,26**	0,576	0,382	0,41	0,23	**0,18**	**0,21**	**211,33**
17	0,566	0,402	0,40	0,25	**0,15**	**0,18**	**178,65**							
**A**	**0,57**	**0,36**					**230,17**	**0,56**	**0,36**					**222,15**
**SD**	**0,11**	**0,12**					**59,51**	**0,07**	**0,08**					**32,83**

Statistical data analysis

Lipids

Larvae

Nomarlity t	RecL	RecBti	Unpaired t test with Welch's correction	
Shapiro-Wilk test			P value	<0,0001
W	0,9675	0,9199	P value sur	****
P value	0,7008	0,0988	Significant	Yes
Passed norı	Yes	Yes	One- or tw	Two-tailed
P value sur	ns	ns	Welch-corı	t=12,25, df=32,29

Adults

Nomarlity t	RecL	RecBti	Unpaired t test with Welch's correction	
Shapiro-Wilk test			P value	<0,0001
W	0,9332	0,9555	P value sur	****
P value	0,1777	0,4591	Significant	Yes
Passed norı	Yes	Yes	One- or tw	Two-tailed
P value sur	ns	ns	Welch-corı	t=5,105, df=32,21

Reducing Sugars

Larvae

Nomarlity '	RecL	RecBti	Mann Whitney test	
Shapiro-Wilk test			P value	0,1082
W	0,8604	0,9141	P value sum	ns
P value	0,0124	0,1172	Significantl	No
Passed norı	No	Yes	One- or two	Two-tailed
P value sur	*	ns	Mann-Whit	104

Adults

Nomarlity t	RecL	RecBti	Unpaired t test with Welch's correction	
Shapiro-Wilk test			P value	0,3063
W	0,9183	0,9601	P value sum	ns
P value	0,0703	0,6645	Significantl	No
Passed norı	Yes	Yes	One- or two	Two-tailed
P value sur	ns	ns	Welch-corr	t=1,046, df=23,67
