# Supplementary material for: Aedes aegypti strain selected with Bacillus thuringiensis svar. israelensis larvicide for 50 generations remains susceptible and exhibited increased fitness
Source: Parasit Vectors. 2025 Oct 7;18:400. doi: 10.1186/s13071-025-07037-x (PMC12506322; doi:10.1186/s13071-025-07037-x)
Supplement: Supplementary file 6 — Additional file 6: Table S6. Dataset of egg viability from an Aedes aegypti strain exposed to Bacillus thuringiensis svar. israelensis for fifty generations (RecBti) compared with a reference strain (RecL). Egg samples were stored between 3 and 180 days after oviposition. Replicates (R) were samples (n > 80 eggs) submerged in water with grass infusion (6 g/L), and first instar larvae were recorded daily until 96 h. [file 13071_2025_7037_MOESM6_ESM.pdf]

Additional file 6: Table S6. Dataset of egg viability from an *Aedes aegypti* strain exposed to *Bacillus thuringiensis* svar. *israelensis* for fifty generations (RecBti) compared to a reference strain (RecL). Eggs samples were stored between 3 and 180 days after oviposition. Replicates (R) were samples (n > 80 eggs) submerged in water with grass infusion (6 g/L) and first instar larvae were recorded daily until 96 h.

**RecBti**								**RecL**							
**Quiescence days**	**Total N eggs**			**24H**		**96H**		**Quiescence days**	**Total N eggs**			**24H**		**96H**	
**3**	**802**	**Replicate**	**N eggs**	**N L1**	**% L1**	**N L1**	**% L1**	**3**	**726**	**Replicate**	**N eggs**	**N L1**	**% L1**	**N L1**	**% L1**
		R1	107	106	99.0	107	100.0			R1	95	50	52.6	82	86.3
		R2	95	85	89.5	94	99.0			R2	98	79	80.6	90	91.8
		R3	97	89	91.8	89	91.8			R3	101	81	80.2	86	85.2
		R4	109	105	96.3	105	96.3			R4	93	82	88.2	83	89.3
		R5	98	96	97.9	98	100.0			R5	82	41	50.0	60	73.2
		R6	95	78	82.1	91	95.8			R6	87	54	62.0	66	75.9
		R7	92	86	93.5	86	93.5			R7	85	58	68.2	80	94.1
		R8	109	105	96.3	105	96.3			R8	85	49	57.7	84	98.8
				**24H**		**96H**						**24H**		**96H**	
**30**	**813**	**Replicate**	**N eggs**	**N L1**	**% L1**	**N L1**	**% L1**	**30**	**786**	**Replicate**	**N eggs**	**N L1**	**% L1**	**N L1**	**% L1**
		R1	89	88	98.8	88	98.8			R1	87	17	19.5	72	82.7
		R2	93	87	93.5	87	93.5			R2	91	26	28.6	75	82.4
		R3	88	37	42.0	88	99.9			R3	106	89	83.9	89	83.9
		R4	90	52	57.8	92	100.0			R4	84	70	83.3	70	83.3
		R5	129	131	100.0	132	100.0			R5	111	103	92.8	105	94.6
		R6	117	116	99.1	116	99.1			R6	92	75	81.5	80	86.9
		R7	106	97	91.5	103	97.2			R7	109	105	96.3	107	98.1
		R8	101	94	93.1	96	95.0			R8	106	98	92.5	104	98.1
**60**	**869**			**24H**		**96H**		**60**	**794**			**24H**		**96H**	
		**Replicate**	**N eggs**	**N L1**	**% L1**	**N L1**	**% L1**			**Replicate**	**N eggs**	**N L1**	**% L1**	**N L1**	**% L1**
		R1	124	107	86.3	119	96.0			R1	103	59	57.3	94	91.2
		R2	105	84	80.0	101	96.1			R2	98	6	6.1	97	98.9
		R3	106	93	87.7	105	99.0			R3	121	76	62.8	81	66.8
		R4	119	88	73.9	119	100.0			R4	92	32	34.8	70	76.1
		R5	99	91	91.9	94	93.9			R5	82	54	65.8	73	89.0
		R6	97	86	88.7	91	93.8			R6	112	98	87.5	106	94.6
		R7	113	107	94.7	108	95.5			R7	85	45	52.9	75	88.1
		R8	106	98	92.5	98	92.5			R8	101	77	76.2	89	88.00
**90**	**861**			**24H**		**96H**		**90**	**904**			**24H**		**96H**	
		**Replicate**	**N eggs**	**N L1**	**% L1**	**N L1**	**% L1**			**Replicate**	**N eggs**	**N L1**	**% L1**	**N L1**	**% L1**

		R1	135	133	98.5	133	98.5			R1	127	104	81.8	107	84.2
		R2	99	92	92.9	93	93.9			R2	121	102	84.2	108	89.2
		R3	103	102	99.0	103	100.0			R3	111	98	88.2	100	90.0
		R4	125	123	98.4	124	99.4			R4	115	107	93	108	93.8
		R5	102	89	87.3	92	90.2			R5	121	111	91.7	112	92.5
		R6	97	82	84.5	83	85.5			R6	98	77	98.6	78	99.6
		R7	91	84	92.3	84	92.3			R7	116	85	73.3	85	73.3
		R8	109	102	93.5	103	94.4			R8	95	79	83.15	99	100.0
**180**	**853**			24H		96H		**180**	**827**			**24H**		**96H**	
		**Replicate**	**N eggs**	**N L1**	**% L1**	**N L1**	**% L1**			**Replicate**	**N eggs**	**N L1**	**% L1**	**N L1**	**% L1**
		R1	105	9	8.5	24	22.9			R1	106	8	7.5	25	23.6
		R2	118	13	11.0	30	25.4			R2	113	11	9.7	25	22.12
		R3	109	5	4.5	9	8.25			R3	99	3	3.0	13	13.13
		R4	103	6	5.8	18	17.47			R4	107	2	1.9	10	9.34
		R5	104	26	25	31	29			R5	97	6	6.2	14	14.4
		R6	95	13	13	19	20			R6	94	18	19.1	23	24.4
		R7	101	21	20	22	21.8			R7	103	8	7.8	11	10.6
		R8	118	22	18	25	21.2			R8	108	16	14.8	17	15.7
**Total Eggs**	**4198**				19			**Total Eggs**	**4037**				#NOME?		
**Average**	**832.3**							**Average**	**807.4**						

Statistical data analysis

**Normality**			**Mann Whitney**			
**Shapiro-Wilk**	**RecLab**	**RecBti**	**Day**	**Time_p**	**p_value**	**significative**
W	0,8908	0,7564	3	24h	0,0014	TRUE
P value	0,2383	0,0096	3	96h	0,0116	TRUE
Passed norm	Yes	No	30	24h	0,1278	FALSE
P value summ	ns	**	30	96h	0,01	TRUE
			60	24h	0,0039	TRUE
			60	96h	0,0181	TRUE
			90	24h	0,0831	FALSE
			90	96h	0,3442	FALSE
			180	24h	0,2271	FALSE
			180	96h	0,3184	FALSE
