# Supplementary material for: Aedes aegypti strain selected with Bacillus thuringiensis svar. israelensis larvicide for 50 generations remains susceptible and exhibited increased fitness
Source: Parasit Vectors. 2025 Oct 7;18:400. doi: 10.1186/s13071-025-07037-x (PMC12506322; doi:10.1186/s13071-025-07037-x)
Supplement: Supplementary file 7 — Additional file 7: Table S7. Dataset of the longevity assay of Aedes aegypti from RecBti and RecL strain, adults stratified by sex. Replicates were groups of 50 adults (1:1 sex ratio) kept with 10% sucrose and water ad libitum. M. Male. F. Female. Two independent assays (1, 2) were done. [file 13071_2025_7037_MOESM7_ESM.pdf]

Additional file 7: Table S7. Dataset of the longevity assay of *Aedes aegypti* from RecBti and RecL strains, adults stratified by sex. Replicates were groups of 50 adults (1:1 sex ratio) kept with 10% sucrose and water ad libitum. M. Male. F. Female.

RecBti

Day	N Male Survival						%a						Total Male	%a	N Female Survival						%a						Total Female	%a	N Total	% a
3	25	25	25	25	25	25	100	100	100	100	100	100	150	**100,00**	25	25	25	25	25	25	100	100	100	100	100	100	150	**100,00**	300	**100,00**
6	25	25	25	25	25	25	100	100	100	100	100	100	150	**100,00**	25	25	25	25	25	25	100	100	100	100	100	100	150	**100,00**	300	**100,00**
11	25	25	25	24	25	25	100	100	100	96	100	100	149	**99,33**	25	25	24	25	25	25	100	100	96	100	100	100	149	**99,33**	298	**99,33**
15	24	24	25	24	24	25	96	96	100	96	96	100	146	**97,33**	25	25	24	25	25	25	100	100	96	100	100	100	149	**99,33**	295	**98,33**
19	24	24	25	24	22	24	96	96	100	96	88	96	143	**95,33**	25	25	24	25	25	25	100	100	96	100	100	100	149	**99,33**	292	**97,33**
22	24	24	23	24	22	24	96	96	92	96	88	96	141	**94,00**	24	25	24	25	25	25	96	100	96	100	100	100	148	**98,67**	289	**96,33**
25	24	24	23	23	20	24	96	96	92	92	80	96	138	**92,00**	24	25	24	25	25	25	96	100	96	100	100	100	148	**98,67**	286	**95,33**
29	21	23	20	23	20	23	84	92	80	92	80	92	130	**86,67**	23	24	24	25	25	25	92	96	96	100	100	100	146	**97,33**	276	**92,00**
32	20	21	19	22	20	23	80	84	76	88	80	92	125	**83,33**	23	24	24	25	25	25	92	96	96	100	100	100	146	**97,33**	271	**90,33**
36	19	19	19	22	20	22	76	76	76	88	80	88	121	**80,67**	23	24	23	25	25	25	92	96	92	100	100	100	145	**96,67**	266	**88,67**
39	18	18	18	20	19	20	72	72	72	80	76	80	113	**75,33**	23	23	22	24	25	25	92	92	88	96	100	100	142	**94,67**	255	**85,00**
43	15	17	16	20	18	18	60	68	64	80	72	72	104	**69,33**	21	22	21	24	25	25	84	88	84	96	100	100	138	**92,00**	242	**80,67**
46	14	17	15	20	17	17	56	68	60	80	68	68	100	**66,67**	20	22	19	24	25	25	80	88	76	96	100	100	135	**90,00**	235	**78,33**
50	12	17	15	19	17	16	48	68	60	76	68	64	96	**64,00**	20	21	19	24	25	24	80	84	76	96	100	96	133	**88,67**	229	**76,33**
53	12	17	14	19	17	16	48	68	56	76	68	64	95	**63,33**	20	19	19	24	25	23	80	76	76	96	100	92	130	**86,67**	225	**75,00**
57	7	10	9	18	17	16	28	40	36	72	68	64	77	**51,33**	14	18	17	24	25	23	56	72	68	96	100	92	121	**80,67**	198	**66,00**
60	1	4	2	16	16	15	4	16	8	64	64	60	54	**36,00**	5	17	16	24	25	23	20	68	64	96	100	92	110	**73,33**	164	**54,67**
64	1	3	1	15	15	11	4	12	4	60	60	44	46	**30,67**	5	14	14	24	24	23	20	56	56	96	96	92	104	**69,33**	150	**50,00**
67	0	3	1	13	15	8	0	12	4	52	60	32	40	**26,67**	5	14	10	23	23	22	20	56	40	92	92	88	97	**64,67**	137	**45,67**
71	0	2	0	13	14	8	0	8	0	52	56	32	37	**24,67**	3	14	8	22	22	21	12	56	32	88	88	84	90	**60,00**	127	**42,33**
74	0	2	0	11	14	7	0	8	0	44	56	28	34	**22,67**	2	13	8	22	19	20	8	52	32	88	76	80	84	**56,00**	118	**39,33**
78	0	2	0	10	14	6	0	8	0	40	56	24	32	**21,33**	2	13	7	22	18	19	8	52	28	88	72	76	81	**54,00**	113	**37,67**
81	0	2	0	10	14	3	0	8	0	40	56	12	29	**19,33**	1	12	6	20	18	17	4	48	24	80	72	68	74	**49,33**	103	**34,33**
85	0	1	0	10	13	3	0	4	0	40	52	12	27	**18,00**	1	8	6	18	16	16	4	32	24	72	64	64	65	**43,33**	92	**30,67**
88	0	1	0	7	11	2	0	4	0	28	44	8	21	**14,00**	1	7	2	15	11	16	4	28	8	60	44	64	52	**34,67**	73	**24,33**
92	0	1	0	5	8	1	0	4	0	20	32	4	15	**10,00**	1	4	0	10	8	14	4	16	0	40	32	56	37	**24,67**	52	**17,33**
95	0	0	0	4	6	0	0	0	0	16	24	0	10	**6,67**	0	2	0	8	7	11	0	8	0	32	28	44	28	**18,67**	38	**12,67**
99	0	0	0	2	2	0	0	0	0	8	8	0	4	**2,67**	0	1	0	6	5	9	0	4	0	24	20	36	21	**14,00**	25	**8,33**
102	0	0	0	0	0	0	0	0	0	0	0	0	0	**0,00**	0	0	0	2	2	7	0	0	0	8	8	28	11	**7,33**	11	**3,67**
106	0	0	0	0	0	0	0	0	0	0	0	0	0	**0,00**	0	0	0	1	0	6	0	0	0	4	0	24	7	**4,67**	7	**2,33**
109	0	0	0	0	0	0	0	0	0	0	0	0	0	**0,00**	0	0	0	0	0	4	0	0	0	0	0	16	4	**2,67**	4	**1,33**
113	0	0	0	0	0	0	0	0	0	0	0	0	0	**0,00**	0	0	0	0	0	3	0	0	0	0	0	12	3	**2,00**	3	**1,00**
116	0	0	0	0	0	0	0	0	0	0	0	0	0	**0,00**	0	0	0	0	0	0	0	0	0	0	0	0	0	**0,00**	0	**0,00**

RecL

N Male Survival					%a					Total Male	% a	N Female Survival					%a					Total Female	%a	N Total	% a
25	25	25	25	25	100	100	100	100	100	**125**	**100,00**	25	25	25	25	25	100	100	100	100	100	125	**100,00**	250	**100,00**
25	25	25	25	25	100	100	100	100	100	**125**	**100,00**	25	25	25	25	25	100	100	100	100	100	125	**100,00**	250	**100,00**
24	23	25	25	25	96	92	100	100	100	**122**	**97,60**	24	25	25	25	25	96	100	100	100	100	124	**99,20**	246	**98,40**
24	22	22	25	25	96	88	88	100	100	**118**	**94,40**	24	25	25	25	25	96	100	100	100	100	124	**99,20**	242	**96,80**
24	22	22	24	25	96	88	88	96	100	**117**	**93,60**	24	25	25	25	25	96	100	100	100	100	124	**99,20**	241	**96,40**
22	22	22	24	25	88	88	88	96	100	**115**	**92,00**	24	25	25	25	25	96	100	100	100	100	124	**99,20**	239	**95,60**
22	20	18	24	23	88	80	72	96	92	**107**	**85,60**	23	25	25	25	25	92	100	100	100	100	123	**98,40**	230	**92,00**
21	18	18	23	19	84	72	72	92	76	**99**	**79,20**	23	25	25	25	25	92	100	100	100	100	123	**98,40**	222	**88,80**
21	18	17	21	19	84	72	68	84	76	**96**	**76,80**	23	25	25	25	25	92	100	100	100	100	123	**98,40**	219	**87,60**
16	14	16	20	16	64	56	64	80	64	**82**	**65,60**	23	24	24	25	25	92	96	96	100	100	121	**96,80**	203	**81,20**
13	10	12	19	13	52	40	48	76	52	**67**	**53,60**	20	18	24	25	22	80	72	96	100	88	109	**87,20**	176	**70,40**
7	3	8	16	11	28	12	32	64	44	**45**	**36,00**	18	5	23	25	6	72	20	92	100	24	77	**61,60**	122	**48,80**
4	3	5	13	9	16	12	20	52	36	**34**	**27,20**	9	4	22	25	6	36	16	88	100	24	66	**52,80**	100	**40,00**
0	3	4	12	7	0	12	16	48	28	**26**	**20,80**	0	4	22	24	4	0	16	88	96	16	54	**43,20**	80	**32,00**
0	0	4	12	5	0	0	16	48	20	**21**	**16,80**	0	3	21	22	4	0	12	84	88	16	50	**40,00**	71	**28,40**
0	0	3	12	3	0	0	12	48	12	**18**	**14,40**	0	1	19	22	4	0	4	76	88	16	46	**36,80**	64	**25,60**
0	0	3	12	1	0	0	12	48	4	**16**	**12,80**	0	0	19	18	4	0	0	76	72	16	41	**32,80**	57	**22,80**
0	0	2	12	1	0	0	8	48	4	**15**	**12,00**	0	0	17	18	3	0	0	68	72	12	38	**30,40**	53	**21,20**
0	0	2	12	1	0	0	8	48	4	**15**	**12,00**	0	0	17	18	3	0	0	68	72	12	38	**30,40**	53	**21,20**
0	0	2	12	1	0	0	8	48	4	**15**	**12,00**	0	0	17	17	3	0	0	68	68	12	37	**29,60**	52	**20,80**
0	0	2	12	1	0	0	8	48	4	**15**	**12,00**	0	0	17	17	3	0	0	68	68	12	37	**29,60**	52	**20,80**
0	0	2	11	1	0	0	8	44	4	**14**	**11,20**	0	0	17	16	3	0	0	68	64	12	36	**28,80**	50	**20,00**
0	0	2	8	0	0	0	8	32	0	**10**	**8,00**	0	0	17	16	3	0	0	68	64	12	36	**28,80**	46	**18,40**
0	0	0	8	0	0	0	0	32	0	**8**	**6,40**	0	0	15	15	3	0	0	60	60	12	33	**26,40**	41	**16,40**
0	0	0	5	0	0	0	0	20	0	**5**	**4,00**	0	0	11	12	3	0	0	44	48	12	26	**20,80**	31	**12,40**
0	0	0	2	0	0	0	0	8	0	**2**	**1,60**	0	0	9	11	3	0	0	36	44	12	23	**18,40**	25	**10,00**
0	0	0	0	0	0	0	0	0	0	**0**	**0,00**	0	0	7	8	2	0	0	28	32	8	17	**13,60**	17	**6,80**
0	0	0	0	0	0	0	0	0	0	**0**	**0,00**	0	0	3	6	2	0	0	12	24	8	11	**8,80**	11	**4,40**
0	0	0	0	0	0	0	0	0	0	**0**	**0,00**	0	0	2	2	1	0	0	8	8	4	5	**4,00**	5	**2,00**
0	0	0	0	0	0	0	0	0	0	**0**	**0,00**	0	0	0	2	0	0	0	0	8	0	2	**1,60**	2	**0,80**
0	0	0	0	0	0	0	0	0	0	**0**	**0,00**	0	0	0	1	0	0	0	0	4	0	1	**0,80**	1	**0,40**
0	0	0	0	0	0	0	0	0	0	**0**	**0,00**	0	0	0	0	0	0	0	0	0	0	0	**0,00**	0	**0,00**
0	0	0	0	0	0	0	0	0	0	**0**	**0,00**	0	0	0	0	0	0	0	0	0	0	0	**0,00**	0	**0,00**

Statistical data analysis

Survival Male and Female

	Statistical By Day								Statistical By Day					
	RecBti x RecL			Normality Test					M RecBti x M RecL			F RecBti x F RecL		
RecL	Day	p_value	Significative		Male Rec	Male R	Female	Female	Day	p_value	Signific	Day	p_value	Significative
	3	ND	ND	Shapiro-Wilk test					3	ND	ND	3	ND	ND
0,92	6	ND	ND	W	0,8815	0,84	0,846	0,862	6	ND	ND	6	ND	ND
0,02	11	0,595	FALSE	P value	0,0022	3E-04	3E-04	7E-04	11	0,415	FALSE	11	1	FALSE
No	15	0,497	FALSE	Passed	No	No	No	No	15	0,622	FALSE	15	1	FALSE
*	19	0,546	FALSE	P value	**	***	***	***	19	0,686	FALSE	19	1	FALSE
	22	0,702	FALSE						22	0,556	FALSE	22	0,724	FALSE
	25	0,302	FALSE						25	0,219	FALSE	25	0,907	FALSE
	29	0,156	FALSE						29	0,158	FALSE	29	0,523	FALSE
	32	0,309	FALSE						32	0,195	FALSE	32	0,523	FALSE
	36	0,043	TRUE						36	0,04	TRUE	36	1	FALSE
	39	0,052	TRUE						39	0,041	TRUE	39	0,307	FALSE
	43	0,035	TRUE						43	0,017	TRUE	43	0,23	FALSE
	46	0,022	TRUE						46	0,008	TRUE	46	0,196	FALSE
	50	0,017	TRUE						50	0,01	TRUE	50	0,166	FALSE
	53	0,017	TRUE						53	0,01	TRUE	53	0,12	FALSE
	57	0,055	TRUE						57	0,035	TRUE	57	0,126	FALSE
	60	0,12	FALSE						60	0,098	FALSE	60	0,12	FALSE
	64	0,119	FALSE						64	0,165	FALSE	64	0,118	FALSE
	67	0,142	FALSE						67	0,268	FALSE	67	0,119	FALSE
	71	0,199	FALSE						71	0,454	FALSE	71	0,14	FALSE
	74	0,233	FALSE						74	0,574	FALSE	74	0,169	FALSE
	78	0,233	FALSE						78	0,574	FALSE	78	0,17	FALSE
	81	0,234	FALSE						81	0,337	FALSE	81	0,199	FALSE
	85	0,234	FALSE						85	0,196	FALSE	85	0,168	FALSE
	88	0,233	FALSE						88	0,196	FALSE	88	0,359	FALSE
	92	0,31	FALSE						92	0,194	FALSE	92	0,645	FALSE
	95	0,454	FALSE						95	0,223	FALSE	95	0,707	FALSE

99	0,513	FALSE	99	0,221	FALSE	99	0,708	FALSE
102	0,922	FALSE	102	ND	ND	102	0,922	FALSE
106	0,223	FALSE	106	ND	ND	106	0,223	FALSE
109	1	FALSE	109	ND	ND	109	1	FALSE
113	0,465	FALSE	113	ND	ND	113	0,465	FALSE
116	ND	ND	116	ND	ND	116	ND	ND
